# S1PR1 induces metabolic reprogramming of ceramide in vascular endothelial cells, affecting hepatocellular carcinoma angiogenesis and progression

**DOI:** 10.1038/s41419-022-05210-z

**Published:** 2022-09-06

**Authors:** Xuehong Wang, Zhidong Qiu, Wei Dong, Zebin Yang, Junnan Wang, Hailiang Xu, Tian Sun, Zhaoquan Huang, Junfei Jin

**Affiliations:** 1 Guangxi Key Laboratory of Molecular Medicine in Liver Injury and Repair, the Affiliated Hospital of Guilin Medical University, 541001 Guilin, Guangxi China; 2 Xiangya Hospital, Central South University, 410008 Changsha, Hunan China; 3 Guangxi Health Commission Key Laboratory of Basic Research in Sphingolipid Metabolism Related Diseases, the Affiliated Hospital of Guilin Medical University, 541001 Guilin, Guangxi China; 4 China‒USA Lipids in Health and Disease Research Center, Guilin Medical University, 541001 Guilin, Guangxi China; 5 Department of General Surgery, Yantian District People’s Hospital, Shenzhen, 518081 Guangdong, China; 6 Department of Pathology, the First Affiliated Hospital of Guangxi Medical University, 530000 Nanning, Guangxi China; 7 Department of Pathology, the Affiliated Hospital of Guilin Medical University, 541001 Guilin, Guangxi China

**Keywords:** Tumour angiogenesis, Cancer metabolism

## Abstract

Angiogenesis is a fundamental process underlying the occurrence, growth and metastasis of hepatocellular carcinoma (HCC), a prevalent tumour type with an extremely poor prognosis due to abundant vasculature. However, the underlying mechanism of angiogenesis in HCC remains largely unknown. Herein, we found that sphingosine-1-phosphate receptor 1 (S1PR1) plays an important role in HCC angiogenesis. S1PR1 was found to be selectively and highly expressed in the blood vessels of HCC tissues compared with those of paratumour tissues. Functionally, high expression of S1PR1 in endothelial cells (ECs) promoted angiogenesis and progression of HCC in vitro and in vivo. Mechanistically, proangiogenic factors (S1P, IL-6, VEGFA) in conditioned medium from HCC cells induced the upregulation of S1PR1 in ECs via the phosphorylation of STAT3 at Y705. Further study also revealed that S1PR1 promotes angiogenesis by decreasing ceramide levels via CerS3 downregulation. Interestingly, we demonstrated that S1PR1 downregulates CerS3 by inducing CerS6 translocation into the nucleus to inhibit CerS3 at the transcriptional level in ECs. In addition, we found that a high concentration of Lenvatinib significantly downregulated the expression of S1PR1 and obviously enhanced S1PR1 knockdown-mediated angiogenesis inhibition, indicating that S1PR1 may be a target by which Lenvatinib combats angiogenesis in HCC. Thus, S1PR1 may be an important target for suppressing angiogenesis in HCC, and inhibiting S1PR1 is a promising approach to antitumor therapy in HCC.

## Introduction

Hepatocellular carcinoma (HCC) is one of the most highly aggressive cancers worldwide and has an extremely poor prognosis. Owing to its highly vascularises characteristic, HCC can easily metastasize to neighbouring and distant sites [1]. Thus, antiangiogenic therapy has shown strong anticancer effects in HCC. However, despite the observed clinical benefits of antiangiogenic drugs such as sorafenib, their clinical application remains limited due to adverse events and drug resistance [2]. Thus, more extensive explorations of new angiogenesis regulators are needed.

Tumour angiogenesis is a complex process involving a variety of molecules in the tumour microenvironment (TME), such as cytokines and inflammatory factors, which affect the proliferation, migration, aggregation and new tube formation of ECs [3, 4]. Cytokines have been shown to induce the transformation of normal ECs into HCC-associated ECs (HAECs) to promote angiogenesis and are considered an important mechanism for tumour neovascularization [5, 6]. Tumour angiogenesis can be regulated by the metabolic reprogramming of ECs and metabolic changes in the tumour microenvironment [7, 8]. As one of the hallmarks of cancer, metabolic reprogramming is characterised by glucose metabolism, lipid metabolism and protein metabolism [9]. It is a self-metabolic regulation process by which malignant tumours adapt and proliferate. Recently, the metabolism of ECs was found to drive angiogenesis via changes in fatty acid oxidation, glycolysis, and glutamine metabolism to impact tumour development [10]. However, the effects of reprogramming sphingolipid metabolism in vascular ECs on malignant tumour angiogenesis remain poorly understood.

Sphingolipid metabolism is a complex process that contributes to carcinogenesis, with ceramide being the central molecule. The anabolism of ceramide includes de novo synthesis, the salvage pathway and sphingomyelin hydrolysis [11]. In the process of ceramide metabolism, many bioactive molecules, such as sphingosine-1-phosphate (S1P) and sphingosine (SPH), are produced. S1P is a regulatory factor affecting vascular growth and maturation and has been shown to promote angiogenesis via its receptors S1PR1-5. S1PR1 was the first S1P receptor identified in human umbilical vein ECs (HUVECs) [12] and was recently reported to have an important role in tumour angiogenesis [13, 14], attracting increasing attention as a new antivascular target. However, the role and underlying mechanism of S1PR1 in HCC angiogenesis remain unclear.

In the salvage pathway of ceramide, SPH is converted into ceramide via ceramide synthases (CerS1-6). CerS1-6 are integral membrane proteins of the endoplasmic reticulum that synthesise ceramides of different fatty acyl chain lengths; for instance, CerS1 and CerS4 generate C18-20 ceramides, CerS5 and CerS6 generate C14-16 ceramides, CerS2 selectively generates C22-24 ceramides and CerS3 mediates the synthesis of very-long-chain C28-32 ceramides with polyunsaturated fatty acid residues [11]. Different ceramide chain lengths have been shown to have different roles in pathological and physiological processes. One study reported that C2-ceramide (artificial synthetic ceramide) inhibited tube formation in HUVECs and inhibited AKT/ERK signalling to arrest angiogenesis [15]. However, whether endogenous ceramide inhibits angiogenesis in mammals has yet to be clarified. In this study, we mainly assessed the role of S1PR1 in vascular ECs of HCC and the underlying mechanism. We found that S1PR1 was upregulated in HCC vascular ECs and promoted angiogenesis and progression of HCC. S1PR1-promoted angiogenesis was related to a decrease in CerS3-dependent ceramide levels, and high S1PR1 expression induced CerS6 translocation into nuclei to inhibit the expression of CerS3 at the transcriptional level. In addition, we found that the level of S1PR1 was decreased under treatment with a high concentration of Lenvatinib and that S1PR1 knockdown-mediated angiogenesis inhibition was enhanced under exposure to Lenvatinib. This new mechanism by which S1PR1 promotes angiogenesis may provide a new strategy for antiangiogenic therapy in HCC.

## Materials and methods

### Tube-formation assay

ECs (90 × 10^3^) treated with or without supernatant of HCC cells or other treatments were suspended in 500 μl of serum-free DMEM and seeded on a 24-well plate precoated with Matrigel. After 6 h, images of network formation were acquired and quantified using a light microscope at ×20 magnification. The polygonal areas formed in the EC network were counted in each field.

### Luciferase reporter assay

Luciferase reporter assays were performed with the Dual Luciferase Assay System (Vazyme) according to the manufacturer’s protocol. Briefly, HEK-293T cells were plated into six-well plates and cotransfected with 1.25 μg of CerS3 promoter-luciferase reporter plasmid, 1.25 μg of CerS6 luciferase reporter plasmid and 0.25 μg of pRL-TK plasmid. S1PR1 siRNA was cotransfected with other plasmids into 293T cells. Lip3000 was used for DNA transfection. 293 T cells were lysed with 1× cell lysis buffer after transfection for 48 h. Then, 20 μl of each lysate was transferred to a 96-well plate and detected with a luminometer using a Dual Luciferase Assay System. For each experiment, the firefly luciferase activity was normalised to the activity of Renilla luciferase (an internal control).

### Animal models

Male nude mice (BALB/c-nu, 4 weeks old) were obtained from Hunan SJA Laboratory Animal Co., Ltd. (Hunan, China). The animals were maintained at room temperature (22–25 °C) and had free access to food and water with a 12 h light/dark cycle. All mice used in the experiments throughout the study exhibited normal health. All the procedures involved in animal experiments were performed following the guide for the Administration of Affairs Concerning Experimental Animals, the national guideline for animal experiments and the Guilin Medical University Institutional Ethical Guidelines for animal experiments (2019-0017). For subcutaneous xenograft studies, 20 mice were randomly divided into four groups (including EC, Huh7, Huh7+EC-NC, Huh7+EC-shS1PR1, 5 mice per group), Huh7 cells (5 × 10^6^ cells) were mixed with ECs-NC (2.5 × 10^6^ cells) or ECs-shS1PR1 (2.5 × 10^6^ cells) at a ratio of 2:1 and implanted subcutaneously into the right flank of the mice with Matrigel (ABW, Shanghai, China, 0827245). To monitor tumour growth, the tumour diameter was measured every 2 or 3 days with a digital calliper. The tumour volume was defined as 0.5 × (longest diameter) × (shortest diameter) × (shortest diameter). To assess whether FB1 (0.4 mg/kg, 0.24 mL/mouse) decreased tumour proliferation by inhibiting ceramide generation, 20 mice were randomly assigned into four groups when xenografts generated by the Huh7 + EC-NC cell mixture reached ~50 mm^3^ and were then administered treatments. At the end of the experiments, tumours dissected from individual mice were immediately fixed in 4% paraformaldehyde.

### Statistical analysis

Quantitative data in this study are presented as the mean ± SD. Analyses were performed using SPSS 20.0 software (SPSS, Inc., USA). Student’s paired *t* test was used to analyse the statistical significance of the differences between HCC tissues and peritumoural tissues. One-way analysis of variance (ANOVA) was applied to determine the statistical significance of the differences among different experimental groups. *P* < 0.05 was considered to indicate statistical significance.

Further details of the Materials and methods are provided in the Supplementary Materials and Methods.

## Results

### S1PR1 is upregulated in the vascular ECs of HCC and is associated with the advanced HCC stage

**Fig. 1:**
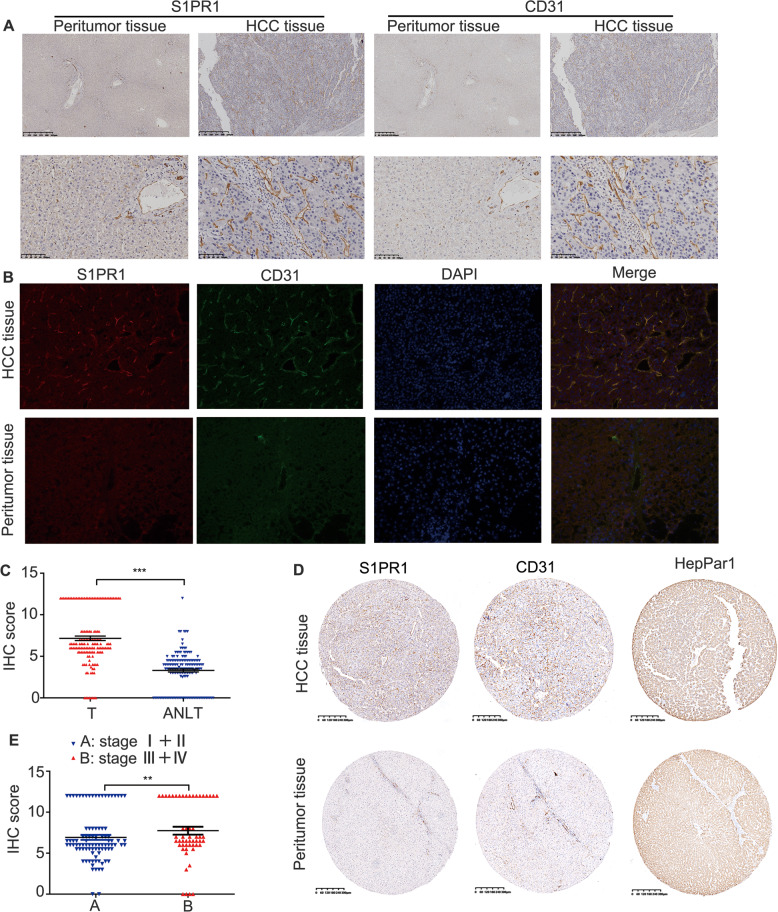
Upregulation of S1PR1 in the vascular ECs of HCC and its association with advanced HCC stage. **A** Serial sections of paraffin-embedded HCC and peritumour tissues were stained with an anti-S1PR1 or an anti-CD31 antibody. Scale bar: 625 μm (top), 100 μm (bottom). **B** Expression of CD31 (green) or S1PR1 (red) in the frozen tissue sections of HCC tissues and peritumour tissues was determined by IF. DAPI staining is indicated in blue. Scale bar: 150 μm. **C** Quantitative analysis of S1PR1 protein levels in 150 paired samples of HCC tissues (T) and paired adjacent nontumorous liver tissues (ANLT) according to IHC scores, and the significant difference was analysed by paired sample Student’s *t* test (***P < 0.01). **D** CD31 (endothelial cell marker) and Hepar-1 (liver cell/liver cancer cell marker) were used to distinguish the expression pattern between endothelial and tumour cells in HCC tissues by IHC. Scale bar: 625 μm. **E** The IHC score was used to analyse the expression of S1PR1 in vascular ECs in different stages of HCC by Student’s *t* test (**P < 0.05).

To explore the role of S1PR1 in HCC angiogenesis, we first examined its in situ expression in serial HCC sections from formalin-fixed paraffin-embedded tissue and frozen tissue. We observed that the expression of S1PR1 in hepatoma cells was lower than that in paratumor liver cells, which was different from the results in another report [16]. However, we also found that S1PR1 was selectively highly expressed on the vascular cells (CD31^+^ cells) of HCC tissues and was negatively or weakly expressed on other stromal tumour cells (Fig. 1A, B). In contrast, S1PR1 expression was lower or undetectable in the hepatic endothelium of peritumoural tissues (Fig. 1A, B). The high expression of S1PR1 on the vascular cells of HCC tissues compared with that on the peritumoural tissue was also confirmed using a tissue microarray (*P* < 0.01) (Fig. 1C). Further experiments using a serial tissue microarray confirmed that S1PR1 was selectively expressed in CD31^+^ cells and was expressed at low levels in hepPar1 (a liver/liver cancer cell marker)-positive cells (Fig. 1D). Next, the clinical relevance of vascular S1PR1 expression in HCC was investigated. Our analysis showed that higher vascular S1PR1 expression was significantly related to a more advanced HCC stage (*P* < 0.05) (Supplementary Table 1, Fig. 1E and Supplementary Fig. S1). These data indicate that S1PR1 is mainly upregulated in the vascular region of HCC tissues and that the vascular expression of S1PR1 could be associated with HCC progression.

### S1PR1 upregulation in HAECs facilitates HCC angiogenesis

**Fig. 2:**
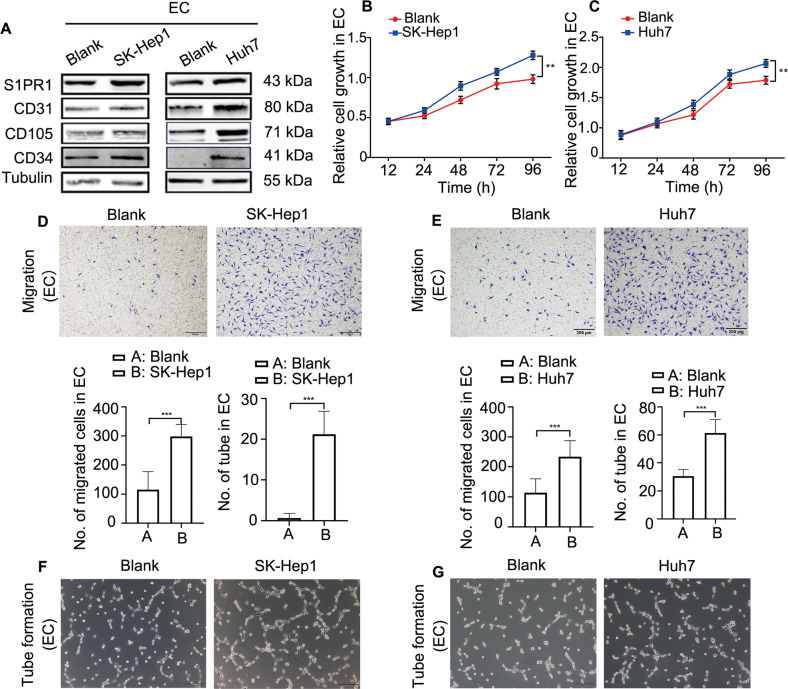
The functions of HAECs induced by conditioned media from HCC cells (SK-Hep1 and Huh7) are enhanced. **A** The markers of ECs CD31/CD34/CD105 and S1PR1 in HAECs induced by conditioned media from SK-Hep1 and Huh7 cells were assessed by WB. **B**, **C** CCK-8 was used to detect the proliferative ability of HAECs induced by conditioned media from SK-Hep1 cells (**B**) and Huh7 cells (**C**). **D**, **E** Migration of HAECs treated with conditioned media from SK-Hep1 cells (**D**) and Huh7 cells (**E**). Scale bar: 200 μm. **F**, **G** Tube formation of HAECs treated with conditioned media from SK-Hep1 cells (**F**) and Huh7 cells (**G**), indicating angiogenic ability. Scale bar: 200 μm.

To investigate the role of S1PR1 in HCC vascular cells, HAECs were established by treating ECs (EA.HY926) cells with conditioned medium from Huh7 or SK-Hep1 cells for 48 h. The characteristics of HAECs treated with conditioned media were validated. As the results showed, the expression of CD31/CD34/CD105 in HAECs was increased significantly, and we also found that S1PR1 expression was upregulated (Fig. 2A). Next, the angiogenic ability of HAECs was examined. The results showed that proliferation (Fig. 2B, C), migration (Fig. 2D, E) and tube-formation capacity (Fig. 2F, G) were all enhanced in HAECs. The major above results were also confirmed in HUVECs, another EC cell line. Under treatment with conditioned medium from SK-Hep1 or Huh7 cells, HUVECs obtained the characteristics of HAECs. The expression of CD31 in HUVECs was increased significantly with the upregulation of S1PR1 (Supplementary Fig. S2A), and migration and tube formation were enhanced (Supplementary Fig. S2B, C).

These data indicated that angiogenesis was promoted in HAECs with S1PR1 upregulation, coinciding with the aforementioned results from Fig. 1.

To further investigate the role of S1PR1 in angiogenesis, siRNA targeting S1PR1 or an S1PR1 overexpression plasmid was used. The results showed that EC migration and tube-formation capacity were increased when S1PR1 was overexpressed (Supplementary Fig. S3A, B), while they were decreased when S1PR1 was knocked down (Supplementary Fig. S3C, D), indicating that S1PR1 plays an important role in the angiogenesis of EC.

### S1PR1 upregulation in HAECs promotes HCC progression

**Fig. 3:**
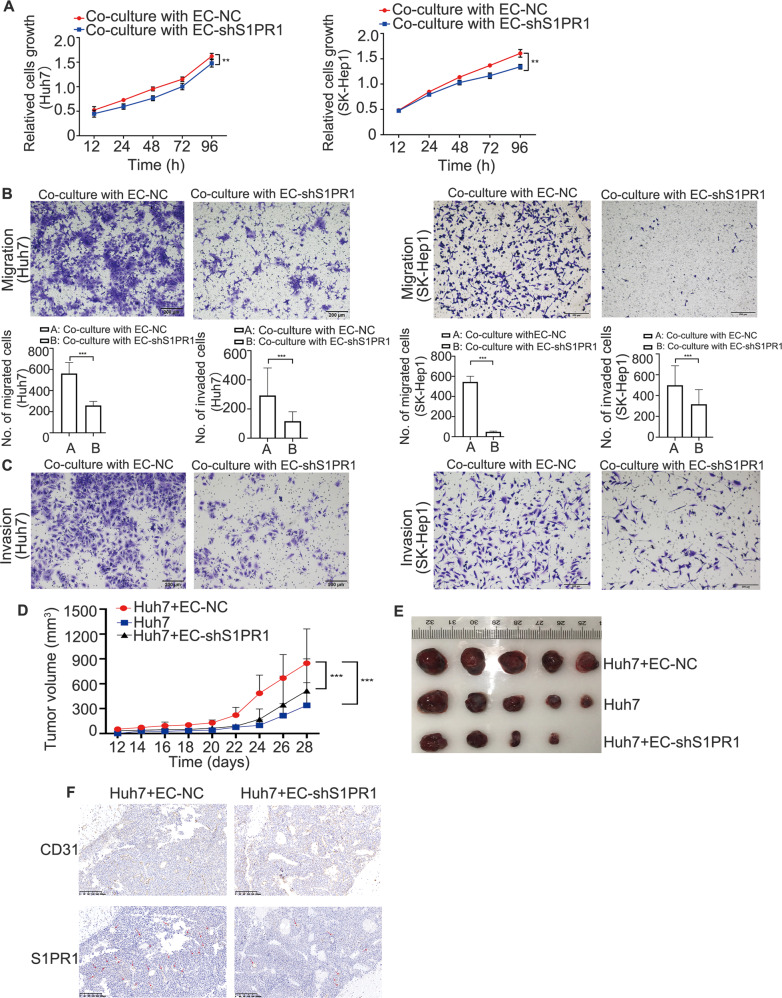
Upregulation of S1PR1 in HAECs promotes the capacities of growth, migration and invasion of HCC cells. **A** CCK-8 assays of HCC cells (Huh7 and SK-Hep1) co-cultured with ECs with S1PR1 knockdown were performed. **B**, **C** The migration and invasion of HCC cells (Huh7 and SK-Hep1) co-cultured with ECs with S1PR1 knockdown were assessed. Scale bar: 200 μm. **D** Growth curves for subcutaneous tumours derived from ECs-NC (2.5 × 10^6^ cells per mouse) or ECs-shS1PR1 (2.5 × 10^6^ cells per mouse) mixed with Huh7 cells (5 × 10^6^ cells per mouse) (*n* = 5 mice per group) and the growth curve of subcutaneous tumours derived from Huh7 cells only (5 × 10^6^ cells per mouse). **E** Representative images of xenografts in **D** at day 28 (*n* = 5 mice per group; there were only four mice that appeared to form liver cancer in the Huh7 + shS1PR1 group). **F** CD31 and S1PR1 expression and localisation were analysed in tumour tissues by immunohistochemistry. Scale bar: 200 μm. Data are presented as the means ± SDs, and an independent *t* test was used to analyse the significant difference. **P < 0.05, ***P < 0.01.

We have demonstrated that high expression of S1PR1 in the vascular region was correlated with the advanced HCC stage. Thus, we further investigated the effects of high S1PR1 expression in HAECs on the proliferation, migration and invasion of HCC cells. Findings from in vitro experiments demonstrated that the proliferation, migration and invasion of Huh7 or SK-Hep1 cells were increased when they were co-cultured with HAECs (Supplementary Fig. S4A–C). To determine whether the level of S1PR1 in EC was associated with these observations, S1PR1 was knocked down in EC co-cultured with Huh7 or SK-Hep1 cells. We observed a significant decrease in the proliferation, migration and invasion of Huh7 or SK-Hep1 cells (Fig. 3A–C). To validate the crosstalk effects between HCC cells and EC cells in vivo, HCC xenografts were generated with the two cell types. We injected EC alone into the left flank and Huh7 alone into the right flank of five mice (Supplementary Fig. S5). We also injected Huh7 cells with EC-NC or Huh7 cells with an EC-shS1PR1 cell mixture at a ratio of 2:1 into the right flank of the other two groups of nude mice. At 28 days after injection, the volume of xenografts from the Huh7 + EC-NC cell mixture group was found to be larger than that from the Huh7 group and the Huh7 + EC-shS1PR1 group (Fig. 3D, E). IHC assessments showed that the number of blood vessels in the tumours generated by Huh7 + ECs-NC was significantly greater than that observed in the tumours generated by Huh7 + ECs-shS1PR1 (Fig. 3F). These observations confirmed that S1PR1 upregulation in HAECs indeed facilitated HCC progression.

### Media from cultured HCC cells induces upregulation of S1PR1 in HAECs via the phosphorylation of STAT3

**Fig. 4:**
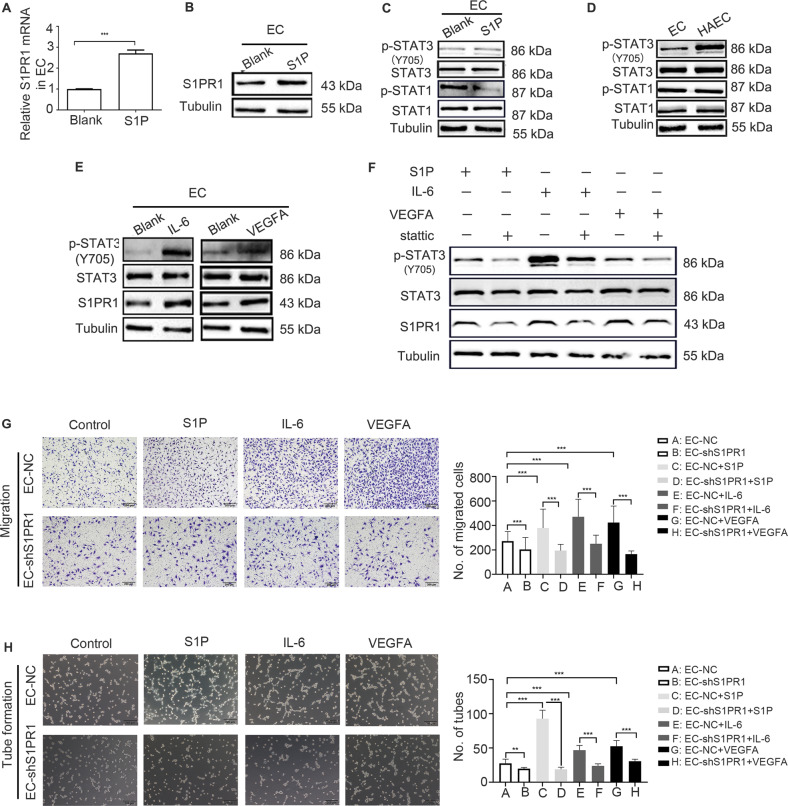
S1PR1 was upregulated in HAECs via the phosphorylation of STAT3, which was stimulated by conditioned medium from HCC cells. **A**, **B** The mRNA (**A**) and protein (**B**) expression of S1PR1 in ECs treated with S1P (40 μM) for 48 h was tested by real-time PCR and WB. **C** STAT1 and STAT3 phosphorylation (Y705) in ECs treated with S1P (40 μM) for 48 h was assessed by WB. **D** STAT1 and STAT3 phosphorylation (Y705) in ECs and HAECs was detected by WB. **E** STAT3 phosphorylation (Y705) in ECs treated with IL-6 (25 ng/ml) or VEGFA (80 ng/ml) for 48 h was assessed by WB. **F** The expression of S1PR1 in HAECs treated with stattic (2 μM) and proangiogenic factors, including IL-6 (25 ng/ml), VEGFA (80 ng/ml) and S1P (40 μM) for 48 h was detected by WB. **G**, **H** The migration (**G**) and tube-formation (**H**) of ECs and ECs with S1PR1 downregulation treated with S1P (40 μM), IL-6 (25 ng/ml) or VEGFA (80 ng/ml) were tested. Scale bar: 200 μm. Data are presented as the means ± SDs, and an independent *t* test was used to analyse the significant difference. ***P < 0.05, **P < 0.01.

From the above experiments, we demonstrated that S1PR1 was increased in HAECs induced by conditioned media from HCC; however, the components of HCC-cultured media inducing S1PR1 upregulation remain unknown. Since it was reported that S1P, as the ligand of S1PR1, could continuously activate S1PR1, leading to S1PR1 overexpression in breast cancer [17], we added S1P to ECs to investigate its effect on S1PR1 expression. Upregulation of S1PR1 (at the mRNA and protein levels) was observed after S1P treatment (Fig. 4A, B). To confirm that S1P secreted from HCC cells induced S1PR1 upregulation in HAECs, we altered the level of SGPL1, a catabolic enzyme of S1P, to change S1P levels in Huh7 or SK-Hep1 cells and their supernatants. First, overexpression of SGPL1 was induced by stably transfecting a cDNA plasmid into SK-Hep1 cells with lower endogenous SGPL1 expression. Knockdown of SGPL1 was achieved by stably transfecting the corresponding shRNA (sh-SGPL1) into Huh7 cells with higher endogenous SGPL1 expression (data not shown). The results from SGPL1-overexpressing SK-Hep1 cells showed that S1P levels were decreased in the cells and the conditioned media, and the data from SGPL1-downregulated Huh7 cells revealed that S1P levels were increased in the cells and the conditioned media (Supplementary Fig. S6A–D). Next, the conditioned medium from HCC cells with SGPL1 overexpression or knockdown was used as the medium for EC culture. We observed that the expression of S1PR1 in EC was upregulated when medium from Huh7 cells with SGPL1 downregulation was used, while the expression of S1PR1 in EC was reduced when medium from SK-Hep1 cells with SGPL1 overexpression was used (Supplementary Fig. S6E, F). Furthermore, EC migration and tube formation were found to be significantly suppressed when EC cells were cultured in conditioned medium from HCC cells with SGPL1 overexpression and enhanced when EC cells were cultured in conditioned medium from HCC cells with SGPL1 knockdown (Supplementary Fig. S6G–J). Based on these results, we deduced that S1P might indeed play an essential role in inducing S1PR1 overexpression in HAECs.

Since it was reported that *STAT3* and *STAT1* are transcription factors affecting S1PR1 gene expression [18, 19], we hypothesised that phosphorylation of STAT3/STAT1 induced S1P-dependent S1PR1 overexpression. First, the association between S1P and STAT3 phosphorylation was investigated. After exposure of ECs to S1P for 48 h, STAT3 phosphorylation at Y705 was increased (Fig. 4C). Second, the level of phosphorylated STAT3/STAT1 in HAECs was detected. The results showed an increase in STAT3 phosphorylation at Y705 in HAECs but no obvious increase in STAT1 phosphorylation (Fig. 4D). These data suggested that some components in the conditioned medium from HCC cells could induce S1PR1 overexpression in HAECs via STAT3 phosphorylation. However, it is well-known that STAT3 phosphorylation could also be stimulated by other factors, such as IL-6 and VEGFA [19, 20], and we also confirmed that the levels of IL-6 or VEGFA were increased in the supernatant of HCC cells compared to that of the control hepatocytes (data not shown). The addition of IL-6 or VEGFA to EC led to an increase in S1PR1 expression and STAT3 phosphorylation at Y705 (Fig. 4E). To further confirm that S1P, IL-6 and VEGFA promoted S1PR1 expression by increasing the phosphorylation level of STAT3 at Y705, stattic, an inhibitor of STAT3 Y705 phosphorylation, was used. The results showed that S1P-, IL-6- and VEGFA-mediated S1PR1 expression could be inhibited by stattic (Fig. 4F). However, under STAT3 knockdown, the expression of S1PR1 appeared to remain unchanged in ECs treated with S1P, IL-6 and VEGFA (Supplementary Fig. S7). These data indicated that S1P, IL-6 and VEGFA upregulated the expression of S1PR1 by phosphorylating STAT3 at Y705. To confirm the role of S1PR1 in EC angiogenesis, S1PR1 was knocked down in ECs treated with S1P, IL-6 and VEGFA. The results revealed that S1P, IL-6 and VEGFA could not significantly increase EC migration and tube formation when S1PR1 was knocked down (Fig. 4G, H). The aforementioned data indicated that S1P is not the only molecule that can upregulate S1PR1 in HAECs via phosphorylation of STAT3 at Y705, and IL-6 and VEGFA have similar effects.

In summary, these findings suggested that S1PR1 upregulation in HAECs was stimulated by some molecules (such as IL-6, VEGFA and S1P) secreted from HCC cells, and the phosphorylation of STAT3 was involved in this process.

### S1PR1 upregulation reduces ceramide levels via inhibition of CerS3 expression

**Fig. 5:**
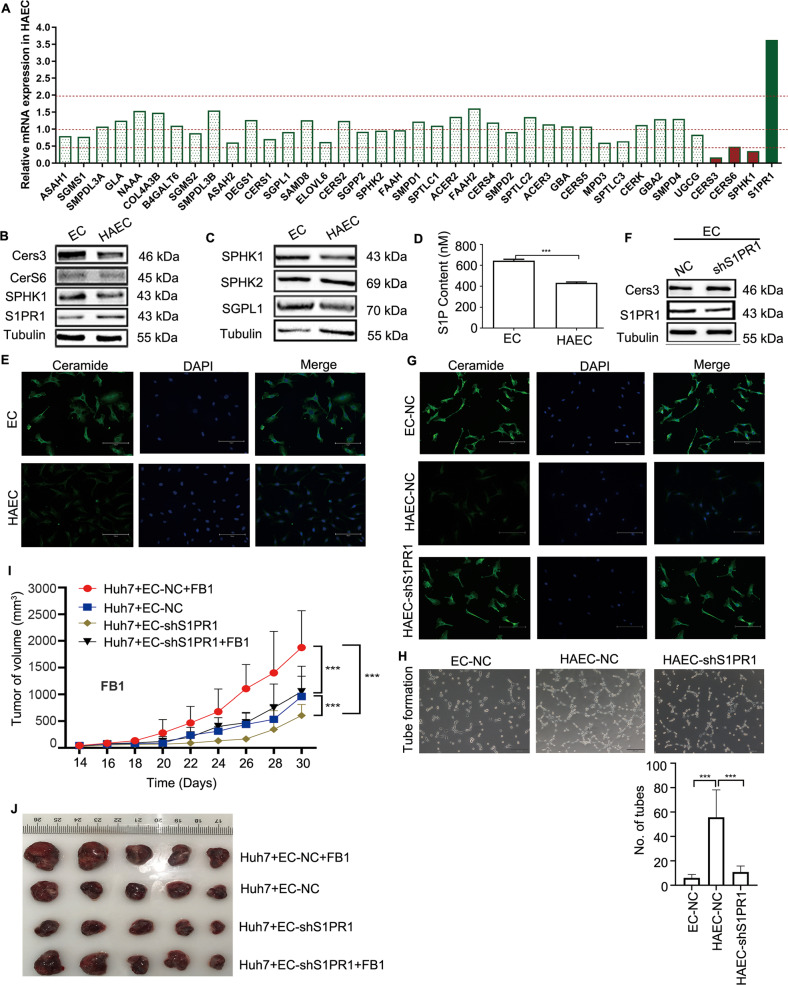
S1PR1 upregulation reduced ceramide levels via inhibition of CerS3 expression. **A** Real-time PCR was used to detect the mRNA expression of S1PR1 in HAECs and investigate the expression of 38 metabolic enzymes of the sphingolipid pathway by PCR array. **B** CerS3/CerS6/SPHK1 in HAECs was assessed by WB. **C** The S1P-related enzymes SPHK1/SPHK2/SGPL1 in HAECs were assessed by WB. **D** The content of S1P in HAECs was measured by ELISA. **E** The level of ceramide (green) in HAECs was determined by IF. DAPI staining is indicated in blue. Scale bar: 150 μm. **F** The expression of CerS3 in ECs-shS1PR1 was detected by WB. **G** IF was used to probe ceramide in HAECs-shS1PR1. Scale bar: 150 μm. **H** Tube formation in HAECs-shS1PR1 was detected. Data are presented as the means ± SDs, and an independent *t* test was used to analyse the significant difference. ***P < 0.01. Scale bar: 200 μm. **I** Growth curve of subcutaneous tumours derived from ECs-NC (2.5 × 10^6^ cells per mouse) or ECs-shS1PR1 (2.5 × 10^6^ cells per mouse) mixed with Huh7 cells (5 × 10^6^ cells per mouse) (*n* = 5 mice per group). These mice were treated with FB1 at 16 days and were stopped by cervical dislocation at 30 days. The other groups of ECs-NC (2.5 × 10^6^ cells per mouse) or ECs-shS1PR1 (2.5 × 10^6^ cells per mouse) mixed with Huh7 cells (5 × 10^6^ cells per mouse) (*n* = 5 mice per group) were used as the control groups. **J** Representative images of xenografts in (**J**) at day 30 (*n* = 5 mice per group).

To investigate the underlying mechanism by which upregulation of S1PR1 in HAECs promotes angiogenesis, a PCR array was used to screen the enzymes in the sphingolipid signalling pathway. Compared to ECs, HAECs had an increase in S1PR1 mRNA expression, as expected, and a decrease in mRNA expression of CerS6 and SPHK1 by more than twofold. It should be noted that CerS3 mRNA was decreased by 74-fold (Fig. 5A), and no significant change in the expression of other enzymes in the sphingolipid signalling pathway was observed (Fig. 5A). Next, we assessed CerS3, CerS6 and SPHK1 protein expression in HAECs (Fig. 5B). Consistent with the change in mRNA, a significant decrease in CerS3 protein expression in HAECs was observed, but no change in CerS6 protein expression was observed (Fig. 5B). Because CerS3, as a ceramide-producing enzyme, was downregulated in HAECs, the ceramide level was examined by immunofluorescence (IF). The IF results showed a significant decrease in ceramide in HAECs (Fig. 5E). When CerS3 was overexpressed, an increase in ceramide was observed (Supplementary Fig. S8A). Then, we explored whether the level of ceramide was regulated by S1PR1. We found that the CerS3 protein (Fig. 5F) and the level of ceramide were increased when S1PR1 was knocked down (Supplementary Figs. S8B and Fig. 5G). These results implied that the S1PR1–CerS3 axis regulated ceramide levels. Although S1PR1 expression affected the tube formation of HAECs (Fig. 5H), the relationship of tube formation with ceramide levels is unknown.

S1P-related enzymes were also detected because S1P is a well-known lipid that promotes angiogenesis. As the results showed, SPHK1 and SGPL1 were downregulated in HAECs, and SPHK2 was not changed (Fig. 5C). To our surprise, S1P level was decreased in HAECs (Fig. 5D), indicating that the angiogenic ability of HAECs was not mainly affected by S1P.

To further study the effect of ceramide on angiogenesis, from day 16 to day 30, FB1 (a potent inhibitor of CerS) was used to treat xenografts generated by the Huh7 + EC-NC or Huh7 + EC-shS1PR1 cell mixture. The results showed that FB1 significantly promoted xenograft development regardless of whether EC S1PR1 was knocked down, indicating that the level of ceramide in tumour tissue affects tumour growth. In addition, EC S1PR1 knockdown could rescue FB1-promoted tumorigenesis, in which S1PR1-dependent EC ceramide was involved (Fig. 5I, J). These in vivo results suggested that ceramide in tumour tissue had an effect on the angiogenesis of HCC and that its level was regulated by EC S1PR1. To further confirm the role of ceramide in angiogenesis, ECs were treated with FB1, and the results showed that FB1 decreased the capacity of EC tube formation accompanied with the reduction in ceramide levels (Supplementary Fig. S9).

### A reduction in ceramide levels activates AKT/ERK signalling to promote angiogenesis by inhibiting PTEN expression

**Fig. 6:**
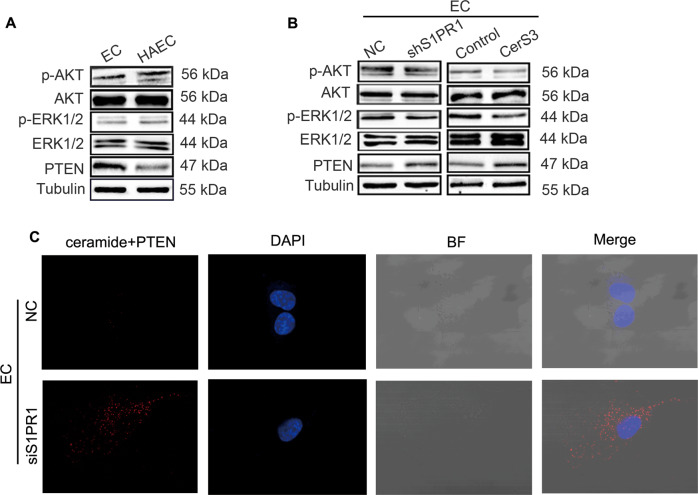
A reduction in ceramide levels and its interaction with PTEN activates AKT/ERK signalling to promote angiogenesis. **A**, **B** p-AKT, AKT, p-ERK, ERK and PTEN expression levels in HAECs (**A**), ECs-shS1PR1 (**B**) and ECs transfected with CerS3 overexpression plasmids (**B**) were determined by WB. **C** An interaction between endogenous ceramide and PTEN was detected by PLA in ECs with S1PR1 knockdown. BF bright field.

We already confirmed that upregulation of S1PR1 reduces ceramide levels in ECs and promotes angiogenesis of HCC. But the mechanism by which the reduction in ceramide promotes angiogenesis remains unknown. As previously reported, C2-ceramide could inhibit angiogenesis by reducing AKT/ERK activities to block the VEGF-VEGFR2 pathway [15, 21], and PTEN was proved to be an inhibited protein for AKT/ERK signalling [22, 23]. Furthermore, C2-ceramide could activate PTEN [24], so we hypothesised that endogenous ceramide might also activate PTEN, leading to a reduction in the inhibitory effect on AKT/ERK1/2 activation and promoting angiogenesis in HAECs. To test this hypothesis, we examined the expression levels of AKT/ERK1/2 and PTEN in HAECs. The results showed a significant increase in AKT and ERK1/2 phosphorylation, indicating that AKT/ERK1/2 is activated in HAECs (Fig. 6A). AKT/ERK1/2 in EC was inhibited when S1PR1 was knocked down (Fig. 6B), suggesting that S1PR1 could regulate AKT/ERK1/2 activation in ECs. Interestingly, CerS3 overexpression in EC led to AKT/ERK1/2 inhibition (Fig. 6B), indicating the involvement of AKT/ERK1/2 in HAEC angiogenesis, which could be regulated by the S1PR1–CerS3–ceramide axis.

Next, we investigated the effect of the S1PR1–CerS3–ceramide axis on PTEN. The results showed that PTEN expression was decreased in HAECs, and it was increased under S1PR1 knockdown or CerS3 overexpression (Fig. 6A, B). Because ceramides can interact with P53, we tried to confirm the interaction between ceramides and PTEN. The PLA results showed that the interaction between ceramides and PTEN in EC was increased when S1PR1 was knocked down (Fig. 6C).

In summary, these results suggested that ceramide reduction could decrease the interaction between ceramide and PTEN, leading to AKT/ERK1/2 activation and promoting angiogenesis in ECs.

### S1PR1-induced nuclear translocation of CerS6 inhibits CerS3 expression at the transcriptional level

**Fig. 7:**
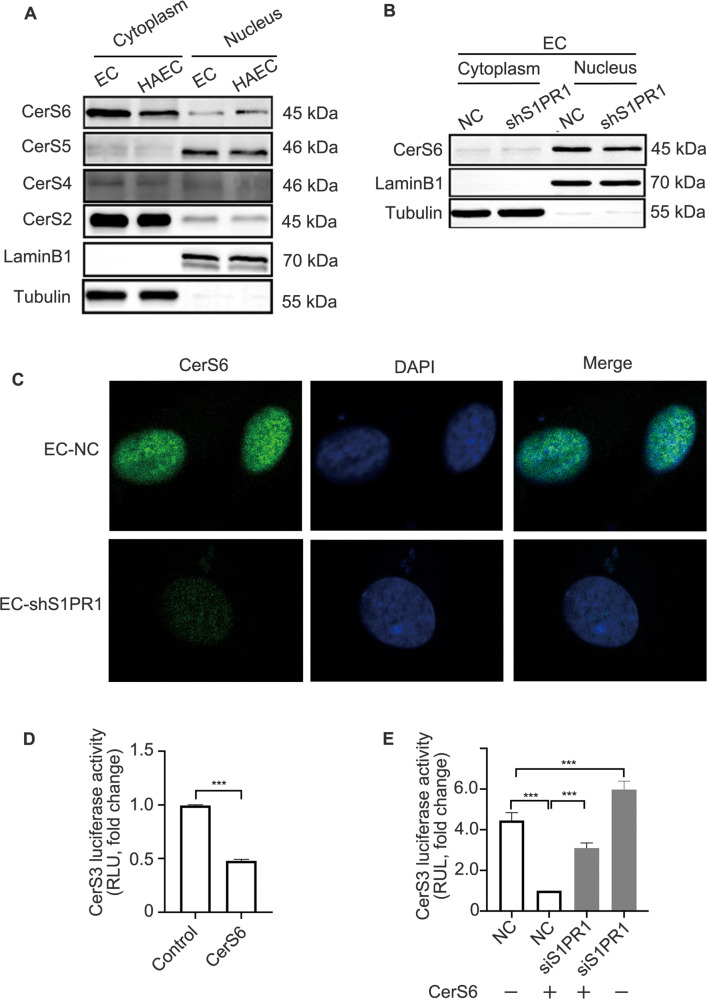
S1PR1-induced nuclear translocation of CerS6 inhibits CerS3 expression at the transcriptional level. **A** The protein expression of CerS2/4/5/6 in the cytoplasm and nucleus of HAECs was detected by WB. **B** The protein expression of CerS6 in the cytoplasm and nucleus of EC-shS1PR1 was assessed by WB. **C** The level of CerS6 in the cytoplasm and nucleus of ECs-shS1PR1 was detected by IF. Scale bar: 50 μm. **D** Luciferase assays with the CerS3 promoter were performed in HK-293T cells transfected with pDNA-CerS6 for 48 h. **E** Luciferase assays with the CerS3 promoter were performed in HK-293T cells pretreated with siS1PR1 for 24 h and then transfected with pDNA-CerS6 for 48 h.

From the above results, it was surprising to find that the protein and mRNA expression of CerS3 were all decreased. This led us to hypothesise that CerS3 could be regulated at the transcriptional level. Studies have shown that all CerSs, apart from CerS1 in mammals, contain a homeodomain that often acts as a transcriptional regulator in the nucleus [25–27]. Therefore, we assumed that there might exist mutual regulation between CerSs. The nuclear localisation signal (NLS) was in the homeodomain of CerSs, except for CerS3 [28], and CerS6 could transcriptionally regulate ASAH1 expression [29], suggesting that CerSs act as transcriptional regulatory factors, so we further hypothesised that CerS3 was regulated at the transcriptional level by other CerSs that could enter the nucleus. The protein expression levels of CerS2, CerS4, CerS5 and CerS6 were assessed, and we found decreased expression of CerS6 protein in the cytoplasm of HAECs but a significant increase in its expression in the nucleus; however, no significant difference in CerS2, CerS4 and CerS5 was observed in HAECs and ECs (Fig. 7A). Interestingly, we observed a reduction in CerS6 expression in the EC nucleus when S1PR1 was knocked down (Fig. 7B, C), implying that CerS6 translocation into the EC nucleus is regulated by S1PR1. These data indicated that high expression of S1PR1 induces CerS6 nuclear translocation.

To investigate whether CerS6 regulates CerS3 expression at the transcriptional level, a luciferase assay was performed. The results revealed a significant twofold decrease in CerS3 promoter activity after transfection with the CerS6 plasmid (Fig. 7D), suggesting that CerS6 inhibits the transcriptional expression of CerS3. Moreover, the results showed that CerS3 promoter activity was obviously increased when S1PR1 was downregulated, and knockdown of S1PR1 partly rescued the decrease in CerS3 promoter activity induced by transfection of the CerS6 plasmid (Fig. 7E). These data suggested that S1PR1 plays a prominent role in regulating the level of ceramides and that CerS6 inhibits CerS3 promoter activity.

### S1PR1 is a potential target mediating the anti-angiogenesis effect of Lenvatinib in HCC

**Fig. 8:**
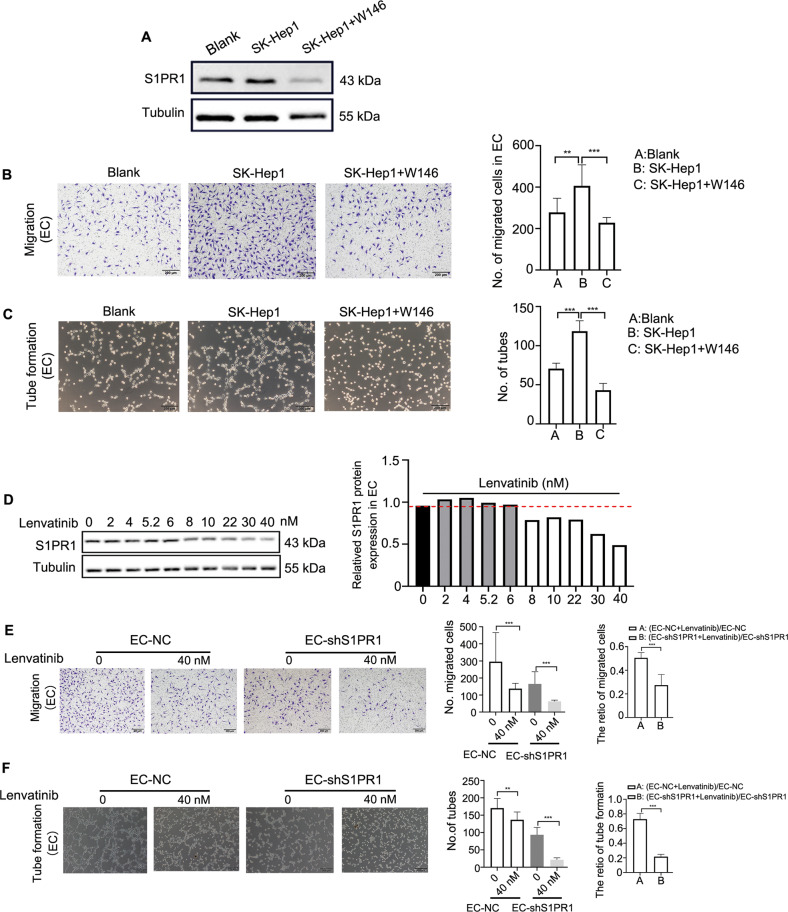
W146 and Lenvatinib target S1PR1 to inhibit angiogenesis. **A** The inhibitory effect of W146 (20 μM) on the promotion of S1PR1 expression induced by the media from SK-Hep1 cells was detected by WB. **B**, **C** The inhibitory effect of W146 (20 μM) on the promotion of migration (**B**) and tube formation (**C**) of ECs induced by media from SK-Hep1 cells was detected. Scale bar: 200 μm. **D** S1PR1 protein expression was tested in ECs treated with Lenvatinib at different concentrations (0–40 nM), and the grey value was calculated by ImageJ. **E**, **F** Migration (**E**, left) and tube formation (**F**, left) of EC-NC, EC-shS1PR1 and EC-shS1PR1 treated with Lenvatinib (40 nM) were tested, and the synergistic effect of Lenvatinib combined with knockdown of S1PR1 on the migration (**E**, right) and tube formation (**F**, left) of EC was analysed. Scale bar: 200 μm.

In this study, we found that S1PR1 might be an important molecular target for HCC angiogenesis, but there are currently no available drugs targeting S1PR1. Therefore, we used W146, which is a selective antagonist of S1PR1, to investigate its antiangiogenic effect. The results showed that W146 inhibited the expression of S1PR1 in EC (Fig. 8A), and W146 also reduced the promotion of migration and tube formation in EC induced by HCC supernatant (Fig. 8B, C), indicating that W146 might be a potential antiangiogenic drug targeting S1PR1 for HCC treatment. Lenvatinib, as a small molecule inhibitor of multiple receptor tyrosine kinases, has been demonstrated to potently block angiogenesis owing to its ability to target multiple receptors, including VEGFR1-3, FGFR1-4, PDGFR, KIT and RET [30–32]. Currently, Lenvatinib is approved for the first-line treatment of patients with unresectable HCC in the USA, EU, Japan and China [33]. However, the relationship between Lenvatinib and S1PR1 has not yet been investigated, and whether S1PR1 is involved in Lenvatinib-mediated angiogenesis blockade remains unknown. Our results showed that a low concentration (2–6 nM) of Lenvatinib did not change the expression of S1PR1. However, as the concentration of Lenvatinib was increased, especially when it reached over 10 nM, a significant decrease in S1PR1 protein expression was observed (Fig. 8D), implying that S1PR1 might be a target of Lenvatinib. Consistent with the aforementioned data, downregulation of S1PR1 alone reduced EC migration and tube formation, but in the presence of 40 nM Lenvatinib, this reduction was significantly enhanced (Fig. 8E, F), indicating that Lenvatinib combined with S1PR1 knockdown had a synergistic antiangiogenic effect.

## Discussion

Tumour angiogenesis is a complex process by which tumours induce the development of new blood vessels to ensure nutritional supply to support increased growth [34]. It has been found that tumours cannot grow beyond 1–2 mm without a vascular supply [35]. Therefore, inhibiting tumour angiogenesis has become a promising antitumor approach, especially for HCC, which often has abundant blood vessels. It was previously found that S1PR1 was expressed abundantly in ECs [12, 36], had angiogenic effects in tumours and was a regulator or the receptor of S1P [5, 13, 14, 20]. Thus, we hypothesised that S1PR1 could be a potential target for antivascular therapy in HCC. First, we assessed the expression of S1PR1 in HCC and found that it was mainly overexpressed in HCC vascular ECs based on co-localisation with CD31. Second, low and high expression of S1PR1 demonstrated opposing effects in in vitro and in vivo experiments, and the knockdown of S1PR1 expression was shown to decrease the density of HCC tumour-bearing mouse tumour vasculature. Third, via mechanistic investigations, S1PR1 was found to regulate the reprogramming of ceramide metabolism in vascular ECs to affect HCC angiogenesis and progression. Fourth, targeting S1PR1 using Lenvatinib or W146 demonstrated encouraging antiangiogenic effects and could be considered for HCC treatment. Importantly, combination of Lenvatinib and HAECs’ S1PR1 knockdown would be a more efficient approach for antiangiogenic treatment in HCC. Taken together, our findings identified S1PR1 as a promising therapeutic target for HCC treatment. However, one study reported that tumour vessels with high expression of S1PR1 (S1PR1 ECTG) showed less angiogenesis, smaller tumours and reduced metastasis; lack of S1PR1 in the vascular endothelium (S1PR1 ECKO) led to more angiogenesis, larger tumours and enhanced lung metastasis; antitumor activities of doxorubicin and anti-PD-1 antibody were more effective in S1PR1 ECTG than in the wild-type counterparts [34]. Their data looked opposite to ours. Tumour cells used for their experiments in this paper including Lewis lung carcinoma cells, B16F10 melanoma cells and breast adenocarcinoma cells E0771, all originated from mouse; but in our study, we used human liver cancer cells. The different type and/or species origin of cells used in these two studies might be the major reason to explain the contradictory results. In addition, all experiments in their study were conducted in mouse models which limited its clinical significance, but in our study, some results were also confirmed in human tissue samples.

The regulation of S1PR1 expression is mediated via several mechanisms, including the important mechanism of S1PR1 transcriptional regulation. Currently, many factors that can directly regulate the activity of the S1PR1 promoter have been identified, such as STAT1[18], STAT3 [19] and Kruppel-like factor 2 (KLF2) [37, 38]. In this study, we mainly investigated the involvement of STAT1 and STAT3 in the regulation of S1PR1 expression in HAECs in HCC. We found that only STAT3 was activated in HAECs, indicating that S1PR1 upregulation might be due to STAT3 activation in HAECs. An increasing amount of evidence suggests that many proangiogenic factors which come from tumour cells, such as S1P, IL-6 and VEGFA, could induce the activation of STAT3 and stimulate the ECs of neighbouring blood vessels [19, 20, 39]. In this study, we revealed that the conditioned medium from HCC cells, containing S1P, IL-6 and VEGFA, induced the overexpression of S1PR1 and promoted angiogenesis of EC via the activation of STAT3; and stattic could block the increasing level of S1PR1 which was induced by S1P, IL-6 and VEGFA, suggesting static may be a potential drug used in HCC antiangiogenic treatment. Interestingly, under STAT3 knockdown, the expression of S1PR1 appeared to remain unchanged in EC treated with S1P, IL-6 and VEGFA. In addition, we found that ECs migration and tube formation mediated by IL-6 and VEGFA were affected not only by VEGFR2 but also by S1PR1, indicating that combined inhibition of VEGFR2 and S1PR1 may be an effective approach for HCC antiangiogenic therapy.

Although many factors have been shown to affect tumour angiogenesis, studies on the role of ceramide in angiogenesis remain largely unknown. In this study, we observed decreased levels of ceramide and reduced tube formation in HAECs; S1PR1 mainly affected the expression of CerS3, while the expression of other CerSs was not significantly affected. Although SPHK1 downregulation resulted in decreased S1P in HAECs, tube formation was still enhanced, suggesting that ceramide alteration could be more effective than S1P alteration in affecting the tube formation of HAECs. We also found that CerS3 had a significant effect on ceramide levels and tube formation in ECs. Downregulation of S1PR1-dependent CerS3 expression decreased ceramide levels and significantly promoted tube formation in ECs. In an in vivo experiment, FB1, a potent inhibitor of CerSs that has been identified to decrease the level of ceramide in many cell types [40], was applied to tumour-bearing mice. In this study, we have proved that FB1 could reduce the level of ceramide in ECs and promote the angiogenesis of ECs in vitro, which is beneficial for the progression of the tumour. In the animal study, we found that the xenografts generated by Huh7+ECs-NC with FB1 grew faster than those generated by Huh7+ECs-NC without FB1, indicating that FB1 can promote the progression of tumours in this model. We also found that FB1 has less effect on the growth of xenografts in the Huh7+ECs-shS1PR1 group compared with Huh7+ECs-NC with FB1 group. These findings suggest that downregulation of S1PR1 in ECs could rescue FB1-induced tumour progression. Notably, the rescue effect of the downregulation of S1PR1 would be associated with the increasing level of ceramide in HAECs. Our in vivo data confirmed that S1PR1 could decrease the level of ceramide to promote angiogenesis.

Mechanistically, we further found that EC S1PR1 could affect the levels of ceramide and PTEN, and downregulation of S1PR1 could increase the interaction between ceramide and PTEN and then inhibit the downstream AKT/ERK signalling. Ceramide, a bioactive signalling sphingolipid, is an important participant in signal transduction. It has been reported that ceramide can combine with proteins to form complexes to strengthen or further weaken the function of proteins. For example, C16-ceramide could activate the p53 tumour suppressor through direct binding in the cellular stress response [41]. In addition, CerS4-generated long-chain ceramides could bind to Smad7 to restrict the TGF-β signalling pathway [42]. These results suggest that ceramide could bind to protein molecules to regulate downstream signalling. In this study, we found that there existed an interaction between ceramide and PTEN. To our knowledge, this is the first time that ceramide has been reported to interact with PTEN. In addition, we found that AKT/ERK1/2 signalling was involved in the angiogenesis of EC and was regulated by CerS3. As reported, AKT signalling is mainly inhibited by PTEN [23], a dual phosphatase with both protein and lipid phosphatase activities [22], and plays an important role in the angiogenesis of glioblastoma [43]. In this study, we found that AKT/ERK1/2 was activated when PTEN was inhibited. Combined with the results showing that AKT/ERK1/2 signalling was inhibited when CerS3 was overexpressed, these findings suggest that AKT/ERK1/2 might be activated due to the weakened interaction between ceramides and PTEN.

Furthermore, we identified that CerS6 acts as a transcription inhibitor to downregulate CerS3. According to the structure of CerSs, ceramide synthase Schlank is not only an enzyme but also a transcriptional regulator in flies [44]. In mammals, except for CerS1, CerSs possess a homeodomain that acts as a transcriptional regulator. Furthermore, CerSs contain the NLSs domain, an important structure for proteins translocating into the nucleus [45]. Therefore, it is reasonable to suggest that some CerS could regulate each other at the transcriptional level. Our results showed that CerS6 could translocate into the nucleus and inhibit the expression of CerS3 at the transcriptional level. Interestingly, CerSs in the same family could regulate each other in the sphingolipid pathway and could be an important way to metabolise sphingolipids in the nuclei.

**Fig. 9:**
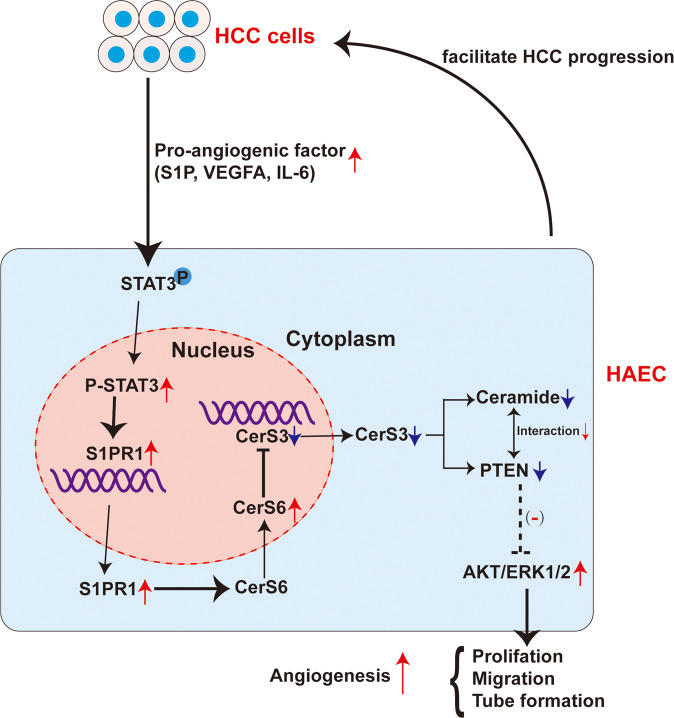
Schematic depiction of the underlying mechanisms of upregulation of S1PR1 in HAECs to promote angiogenesis in HCC. The conditioned medium from HCC cells, containing S1P, IL-6 and VEGFA, induced the overexpression of S1PR1 via the activation of STAT3; overexpression of S1PR1 controls CerS6 translocation into the nucleus of ECs and then inhibits CerS3 expression at the transcriptional level, lead to a decrease in ceramide and PTEN levels, then reduces the interaction between ceramide and PTEN and activates AKT/ERK signalling to promote angiogenesis. Besides, the promotion of angiogenesis in HAECs facilitates HCC progression.

In conclusion, findings from this study confirmed that S1PR1 was upregulated in the vascular ECs of HCC and support it as a novel potential antiangiogenic factor and potential target of Lenvatinib for HCC treatment. Mechanistic studies showed that S1PR1 could be upregulated via STAT3 activation. To our knowledge, this is the first study to reveal that S1PR1 reduces ceramide levels by controlling CerS6 translocation into the nucleus of ECs and then regulates CerS3 expression at the transcriptional level to induce angiogenesis and HCC progression (Fig. 9).

## Supplementary information

Supplementary Materia

Figure S1

Figure S2

Figure S3

Figure S4

Figure S5

Figure S6

Figure S7

Figure S8

Figure S9

Original Data File

aj-checklist-CDDIS

## Data Availability

All data are available in the main text or Supplementary Materials.
