# Supplementary material for: S1PR1 induces metabolic reprogramming of ceramide in vascular endothelial cells, affecting hepatocellular carcinoma angiogenesis and progression
Source: Cell Death Dis. 2022 Sep 6;13(9):768. doi: 10.1038/s41419-022-05210-z (PMC9448762; doi:10.1038/s41419-022-05210-z)
Supplement: Supplementary file 1 — Supplementary Materia [file 41419_2022_5210_MOESM1_ESM.docx]

**Supplementary Material**

**S1PR1 induces metabolic reprogramming of ceramide in vascular endothelial cells, affecting hepatocellular carcinoma angiogenesis and progression**

Xuehong Wang^1,2,3,4*^, Zhidong Qiu^1,2,3,4,5*^, Wei Dong^1,2,3,4^, Zebin Yang^1,3,4^, Junnan Wang^1,3,4^, Hailiang Xu^1,3,4^, Tian Sun^1,3,4^, Zhaoquan Huang^6,7#^, Junfei Jin^1,3,4#^

^1^ Guangxi Key Laboratory of Molecular Medicine in Liver Injury and Repair, the Affiliated

Hospital of Guilin Medical University, Guilin, 541001, Guangxi, China

^2^ Xiangya Hospital, Central South University, Changsha, 410008, Hunan, China

^3^ Guangxi Health Commission Key Laboratory of Basic Research in Sphingolipid Metabolism

Related Diseases, the Affiliated Hospital of Guilin Medical University, Guilin, 541001, Guangxi, China

^4^ China‒USA Lipids in Health and Disease Research Center, Guilin Medical University, Guilin, 541001, Guangxi, China

^5^ Department of General Surgery, Yantian District People's Hospital, Shenzhen, 518081, Guangdong, China

^6^ Department of Pathology, the First Affiliated Hospital of Guangxi Medical University, Nanning, 530000, Guangxi, China

^7^ Department of Pathology, the Affiliated Hospital of Guilin Medical University, Guilin, 541001, Guangxi, China

^#^ Correspondence to Junfei Jin ([junfeijin@glmc.edu.cn](mailto:junfeijin@glmc.edu.cn)), Zhaoquan Huang ([gxlzzq@163.com](mailto:gxlzzq@163.com)), Guangxi Key Laboratory of Molecular Medicine in Liver Injury and Repair, the Affiliated Hospital of Guilin Medical University, Guilin, 541001, Guangxi, China; Tel.: +86 773 2862270; Fax: +86 773 2810411; address: 15 Lequn Road, Guilin, 541001, Guangxi, China.

***** Both authors contributed equally to this work.

**Running title:** S1PR1 promotes hepatocellular carcinoma angiogenesis

**Keywords:** S1PR1, HCC, angiogenesis, ceramide metabolism reprogramming, CerS3

**Supplementary materials and methods**

**1. Biochemical reagents**

Fumonisin B1 (FB1), stattic, IL-6 protein, Lenvatinib and W146 were purchased from MedChemExpress (Monmouth Junction, NJ). Sphingosine-1-phosphate (d18:1, S1P) was purchased from Avanti Polar Lipids (Alabama, USA). VEGFA (VEGF 165 protein) was purchased from Sino Biological (Beijing, China). A sphingosine-1-phosphate ELISA kit was purchased from Echelon Bioscience Inc. (Salt Lake, USA). Duolink® In Situ PLA ® Probe Anti-Rabbit PLUS was purchased from Sigma (USA). The primary antibodies included anti-S1PR1 (Abcam, ab23386, ab137467), anti-CD31 (Abcam, ab28364, ab9498), anti-CD34 (Abcam, ab81289), anti-CD105 (Abcam, ab170943), anti-SPHK1 (Abcam, ab260073), anti-SPHK2 (Abcam, ab215750), anti-PTEN (Abcam, ab170941), anti-SGPL1 (Abcam, ab246929), anti-p-STAT3 (Y105) (CST, 9145), anti-STAT3 (CST, 9139), anti-p-STAT1 (Abcam, ab109457), anti-STAT1 (Abcam, ab92506), anti-p-ERK (CST), anti-ERK (CST), anti-p-AKT (Proteintech), anti-AKT (Proteintech), anti-CerS2 (Novus, NBP1-84537), anti-CerS3 (Novus, NBP1-84536), anti-CerS4 (Novus, NBP1-55731), anti-CerS5 (Novus, NBP1-76964), and anti-CerS6 (Novus, NBP1-92068).

**2. Human samples**

The procedures were approved by the Ethics Board at Guilin Medical University (approval no. 2019QTZCLL-1). Primary HCC tumours and adjacent peritumoural tissues along with clinicopathological information were collected from HCC patients after surgical resection at the Affiliated Hospital of Guilin Medical University, Guilin, Guangxi, China, between March 2019 and December 2020. Clinical samples were collected from patients after obtaining informed consent in accordance with a protocol approved by the Ethics Committee of the Affiliated Hospital of Guilin Medical University (Guilin, China). All fresh tumour tissues were embedded in OCT, immediately snap-frozen in liquid nitrogen and kept at –80°C until use. The HCC tissue microarray (TMA) was obtained from Shanghai Outdo Biotech Co., Ltd. (Shanghai, China).

**3. Immunohistochemistry**

Paraffin-embedded tissues were first heated at 60°C overnight, and the TMA was heated at 60°C for 2 h; then the samples were dewaxed using dimethylbenzene and rehydrated with a gradient series of decreasing concentrations of alcohol. Next, the samples were boiled in citrate antigenic retrieval buffer (pH = 9.0) for 2.5 min using a pressure cooker and cooled to room temperature. Then, endogenous peroxidase activity was quenched with 3% H_2_O_2_ for 10 min at room temperature, and the samples were washed in phosphate-buffered saline (PBS) containing 1% Tween. After that, the sections were incubated with rabbit polyclonal S1PR1 antibody diluted in working solution (1:150, ab23386) or CD31 antibody diluted in 1:200 (ab28364) or HepPar1 (MXB biotechnology, Fuzhou, China) at 4°C overnight, washed in phosphate-buffered saline (PBS) containing 1% Tween, and incubated with secondary antibody (ZSGB–BIO, Beijing, China) at room temperature for 1 h. The staining of the sections was visualized with 3,3-diaminobenzidine tetrahydrochloride (DAB), and the sections were counterstained with haematoxylin. As a negative control, the primary antibody was replaced by nonspecific rabbit serum under the same experimental conditions. The expression levels of S1PR1 protein in the samples were independently evaluated by a semiquantitative IHC method by two pathologists who were blinded to the clinical and follow-up data of the HCC patients. The proportion of tumour cells was scored as follows: grades 0 to 4 (0, no positive cells; 1, < 25% positive cells; 2, 25%-50% positive cells; 3, 50%- 75% positive cells; 4,＞75% positive cells).

**4. Immunofluorescence**

Tumours harvested from HCC patients were protected in tissue protection solution. These tissues were then fixed OCT immediately. Tissues were cryo-sectioned (6 μm thickness). Sections were fixed using precooled acetone for 10 min, blocked in H_2_O_2_ for 10 min and then blocked in 1% BSA for 30 min. Sections were incubated using S1PR1 (ab137467), CD31 (ab9498) primary antibodies followed by appropriate secondary antibodies and DAPI. Images were analysed using a fluorescence microscope.

A suitable quantity of cells was plated into 24-well plates for 24 h. The cells were fixed with 4% PFA for 10 min, permeabilized with 0.1% Triton X-100 for 15 min and then stained with a ceramide (sigma) or CerS6 antibody followed by incubation with the appropriate secondary antibodies and DAPI. Images were analysed using a fluorescence microscope.

**5. Cell culture**

The EA.HY926 cells (ECs) and Huh7 cells were obtained from the Cell Bank of the Chinese Academy of Science (Shanghai, China), and SK-Hep1 and HEK-293T cells were obtained from ATCC. HUVECs were purchased from Meisen CTCC (Zhejiang, China). These cell lines were authenticated by short tandem repeat (STR) validation analysis during the study period. EC, SK-Hep1, Huh7 and HEK-293T cells were cultured in DMEM (Gibco, Thermo Fisher Scientific, MA, USA) supplemented with 10% FBS (Gibco, Thermo Fisher Scientific, MA, USA) and 1% penicillin and streptomycin (Sigma‒Aldrich, St Louis, MO, USA) at 37°C in a humidified atmosphere of 5% CO2. HUVECs were cultured with endothelial cell medium (ScienCell, San Diego, USA, 1001) supplemented with 5% FBS (ScienCell, 0025) and 0.03 mg/mL endothelial cell growth supplement (ECGS, ScienCell, 1052). HUVECs at passages 2-7 were used in all experiments.

When HCC cells reached 60-80% confluence, fresh medium was replenished for 24 h of incubation, and then the supernatant was collected, centrifuged and stored at -20°C. After reaching 60-80% confluence, ECs were induced with conditioned medium (60% HCC supernatant and 40% fresh medium) for 48 h to form HAECs. In this study, S1P (40 μM), IL-6 (25 ng/ml), VEGFA (80 ng/ml), stattic (2 μM), W146 (20 μM) and FB1 (0.1 μM) were used to treat ECs for 48 h.

**6. Plasmid transfection and RNA interference**

Full-length S1PR1-3Flag and full-length CerS3-3Flag were inserted into the pcDNA3.1 vector (all purchased from GenePharma, Jiangsu, China). Each experimental plasmid was expressed in cells using Lipofectamine 3000 reagent (Invitrogen) according to the manufacturer’s instructions. To knock down the target gene, small-interfering RNA oligonucleotides targeting S1PR1 and STAT3 (GenePharma, Jiangsu, China) were transfected into EC cells using Lipofectamine 3000 reagent (Invitrogen) according to the manufacturer’s protocol. The following sequences were used: S1PR1 siRNA sense: 5’-GCAAAUUCAAGCGACCCAUTT-3’, anti-sense: 5’-AUGGGUCGCUUGAAUUUGCTT-3’. STAT3 siRNA sense: 5’-GCAACAGAUUGCCUGCAUUTT-3’, anti-sense: 5’-AAUGCAGGCAAUCUGUUGCTT-3’.

**7. Construction of stable cell lines**

To obtain cell lines stably expressing low levels of S1PR1, ECs were transfected with shRNA-S1PR1 and sh-control viruses (Genechem, Shanghai, China). The infection efficiency was confirmed by qRT‒PCR. S1PR1-shRNA: 5’-GCAAATTCAAGCGACCCATCA-3’.

**8. Western blotting**

After transfection or other treatments, cells were lysed in RIPA lysis buffer (Beyotime) containing 1% protease inhibitor PMSF (100 mM) and 1% phosphatase inhibitors. Cell lysates were then centrifuged at 12000 rpm for 10 min at 4 °C. The supernatant was harvested, and the protein concentration was determined using the Pierce BCA protein assay kit (Beyotime). The protein was then mixed with SDS‒PAGE Sample Loading Buffer 4X (Solarbio, Beijing, China) and boiled for 5 min. An equivalent amount of protein was separated by SDS‒PAGE and transferred to polyvinyl difluoride membranes (Millipore, Billerica, MA, USA). The membranes were blocked with 5% (w/v) nonfat milk in TBST (1 M Tris buffer saline, pH 8.0, 5 M NaCl, 0.1% Tween-20) and shaken lightly for 1 h at room temperature. The membranes were then washed three times and incubated with primary antibodies overnight at 4 ℃ followed by incubation with secondary anti-rabbit or mouse IgG antibodies conjugated with HRP for 1 h at 37 °C. Signals were detected using chemiluminescence reagent.

The cytoplasmic and nuclear fractions of ECs were obtained using the Cytoplasmic and Nuclear Fractionation kit from Invent Biotechnologies Inc.

**9. Real-time PCR**

Total RNA was isolated from cells using an RNA extraction Kit from Vazyme (Nanjing, China). First-strand complementary DNA (cDNA) was synthesized from 1 μg of total RNA using the Fast-Quant RT Kit (with gDNase). The board of the PCR array was purchased from QIAGEN. The following primers were used: S1PR1 forward, 5’- CACAACGGGAGCAATAAC-3’, reverse, 5’-AAGACGCTCAGGACGATA-3’; GAPDH forward, 5’-CAGGAGGCATTGCTGATGAT-3’, reverse, 5’-GAAGGCTGGGGCTCATTT-3’. Real-time PCR was performed in duplicate a total of three times using 25 μl of reaction mixture containing 2.0 μl of cDNA, 1 μM primers, and 12.5 μl of TB Green Premix. The relative gene expression levels were quantified by first using the comparative CT method and then normalizing the value for each target gene to that of GAPDH; the results were expressed as the fold change in expression compared to that of the control.

The PCR array was purchased from QIAGEN (Duesseldorf, Germany). All steps were carried out according to the instructions.

**10. Cell viability assay**

After transfection, EA.HY926 cells (6 × 10^3^/well) or HCC cells (5 × 10^3^/well) were seeded onto 96-well plates and cultured for 12/24/48/72/96 h. Finally, cytotoxicity was assessed using the Cell Counting Kit-8 (CCK-8; DOJINDO, Shanghai, China). CCK-8 reagent was added to the cells at 37°C for 1 h, and the optical density was measured by a microplate reader set at 450 nm.

**11. Migration and invasion assay**

Cell migration and invasion assays were performed using 8-μm pore size Transwell chambers (Corning, USA). In brief, 2-5×10^4^ cells were cultured in the upper chamber with serum-free medium, and the lower chamber was filled with DMEM containing 10% FBS. After 48 h of incubation, the adherent cells in the inner surface of the chamber were wiped with a cotton swab. The migrated or invaded cells were fixed with 4% paraformaldehyde for 30 min, stained with 0.1% crystal violet overnight, and then photographed with an inverted microscope.

**12. Enzyme-Linked Immunosorbent Assay (ELISA)**

S1P in medium or cell lysate was quantified using competitive ELISA kits according to the manufacturer’s protocol. The cells were lysed via serum-free repeated freezing and thawing in liquid nitrogen 5 times.

**13. Proximity ligation assay (PLA)**

The interaction between endogenous ceramide and PTEN in situ was detected by PLA in EC cells according to the manufacturer’s instructions and a previous report with slight modification(1).

**Supplementary Tables and Figures**

**Table 1: Correlation of S1PR1 expression with clinicopathological features in HCC**

| **Variables** | **Expression of S1PR1 in HAECs** | | **χ^2^** | **P value** |
| --- | --- | --- | --- | --- |
|  | **Low expression** | **High expression** |  |  |
| **Age** |  |  | 1.594 | 0.207 |
| ≤60 | 20 | 91 |  |  |
| ＞60 | 10 | 26 |  |  |
| **Sex** |  |  | 2.088 | 0.149 |
| Female | 22 | 99 |  |  |
| Male | 8 | 18 |  |  |
| **Tumour size (cm)** |  |  | 0.139 | 0.710 |
| ≤ 5 | 7 | 29 |  |  |
| > 5 | 20 | 69 |  |  |
| **Differentiation** |  |  | 0.724 | 0.395 |
| I~II | 25 | 89 |  |  |
| II~III | 5 | 28 |  |  |
| **TNM stage** |  |  | 3.92 | 0.048 |
| I~II | 13 | 74 |  |  |
| III~Ⅳ | 17 | 43 |  |  |
| **AFP (ng/mL)** |  |  | 0.479 | 0.489 |
| ≤ 200 | 17 | 56 |  |  |
| > 200 | 13 | 57 |  |  |
| **CEA (ng/mL)** |  |  | 0.438 | 0.508 |
| ≤ 3.4 | 19 | 65 |  |  |
| > 3.4 | 7 | 17 |  |  |
| **HBsAg (ng/mL)** |  |  | 0.076 | 0.782 |
| ≤ 0.5 | 5 | 23 |  |  |
| > 0.5 | 23 | 91 |  |  |
| **Cirrhosis** |  |  | 0.842 | 0.359 |
| Yes | 14 | 65 |  |  |
| No | 16 | 51 |  |  |
| **Metastasis** |  |  | 0.208 | 0.648 |
| Yes | 20 | 83 |  |  |
| No | 10 | 34 |  |  |

**Table 2. Array data for differentially expressed mRNAs in HAECs**

| **Gene**  **symbol** | **Protein** | **Functions** | **Fold change**  **(HAECs/ECs)** |
| --- | --- | --- | --- |
| ASAH1 | Acid Ceramidase | Hydrolyzes ceramide into sphingosine and free fatty acid | 0.798833 |
| SGMS1 | Sphingomyelin Synthase 1 | synthesize sphingomyelin | 0.77497 |
| SMPDL3A | Sphingomyelin Phosphodiesterase Acid Like 3A | Has in vitro nucleotide phosphodiesterase activity with nucleoside triphosphates | 1.077526 |
| GLA | Galactosidase Alpha | Catalyzes the hydrolysis of glycosphingolipids | 1.250108 |
| NAAA | N-Acylethanolamine Acid Amidase | Degrades fatty acid amides to their corresponding acids | 1.539458 |
| COL4A3B | Ceramide Transporter 1 | Mediates the intracellular trafficking of ceramides | 1.48646 |
| B4GALT6 | Beta-1,4-Galactosyltransferase 6 | Catalyzes the synthesis of lactosylceramide | 1.101432 |
| SGMS2 | Sphingomyelin Synthase 2 | contributes to sphingomyelin synthesis | 0.8844743 |
| SMPDL3B | Sphingomyelin Phosphodiesterase Acid Like 3B | Lipid-modulating phosphodiesterase | 1.548491 |
| ASAH2 | N-Acylsphingosine Amidohydrolase 2 | hydrolyzes ceramides into sphingosine and free fatty acids at neutral pH | 0.6086911 |
| DEGS1 | Delta 4-Desaturase, Sphingolipid 1 | Converts D-erythro-sphinganine to D-erythro-sphingosine | 1.267403 |
| CERS1 | Ceramide Synthase 1 | catalyzes the transfer of the acyl chain from acyl-CoA to a sphingoid base | 0.7109022 |
| SGPL1 | Sphingosine-1-Phosphate Lyase 1 | Cleaves phosphorylated sphingosine-1-phosphate into fatty aldehydes and phosphoethanolamine | 0.9174014 |
| SAMD8 | Sphingomyelin Synthase-Related Protein 1 | Synthesize Sphingomyelin through transfer a phosphatidyl head group on to the primary hydroxyl of ceramide | 1.261909 |
| ELOVL6 | ELOVL Fatty Acid Elongase 6 | Catalyzes the first and rate-limiting reaction of the four reactions that constitute the long-chain fatty acids elongation cycle | 0.6207693 |
| CERS2 | Ceramide Synthase 2 | Ceramide synthase that catalyzes the transfer of the acyl chain from acyl-CoA to a sphingoid base, with high selectivity toward very-long-chain fatty acyl-CoA | 1.241032 |
| SGPP2 | Sphingosine-1-Phosphate Phosphatase 2 | Has specific phosphohydrolase activity towards sphingoid base 1-phosphates | 0.9246123 |
| SPHK2 | Sphingosine Kinase 2 | Catalyzes the phosphorylation of sphingosine to form sphingosine-1-phosphate | 0.9580656 |
| SPHK1 | Sphingosine Kinase 1 | Catalyzes the phosphorylation of sphingosine to form sphingosine 1-phosphate | 0.3554419 |
| FAAH | Fatty Acid Amide Hydrolase | Catalyzes the hydrolysis of endogenous amidated lipids to their corresponding fatty acid | 0.9673892 |
| SMPD1 | Sphingomyelin Phosphodiesterase 1 | Converts sphingomyelin to ceramide | 1.224832 |
| SPTLC1 | Serine Palmitoyltransferase Long Chain Base Subunit 1 | Serine palmitoyltransferase (SPT) | 1.103057 |
| ACER2 | Alkaline Ceramidase 2 | Catalyzes the hydrolysis of ceramides into sphingoid bases like sphingosine and free fatty acids at alkaline pH | 1.363426 |
| FAAH2 | Fatty Acid Amide Hydrolase 2 | Catalyzes the hydrolysis of endogenous amidated lipids to their corresponding fatty acids | 1.611637 |
| CERS4 | Ceramide Synthase 4 | Ceramide synthase that catalyzes formation of ceramide from sphinganine and acyl-CoA substrates, with high selectivity toward long and very-long chains (C18:0-C22:0) as acyl donor | 1.19633 |
| SMPD2 | Sphingomyelin Phosphodiesterase 2 | Catalyzes the hydrolysis of sphingomyelin to form ceramide and phosphocholine | 0.9194998 |
| SPTLC2 | Serine Palmitoyltransferase 2 | Serine palmitoyltransferase (SPT) | 1.355317 |
| ACER3 | Alkaline Ceramidase 3 | Catalyzes the hydrolysis of unsaturated long-chain C18:1-, C20:1- and C20:4-ceramides, dihydroceramides and phytoceramides into sphingoid bases at alkaline pH | 1.146424 |
| GBA | Glucosylceramidase Beta | Catalyzes, within the lysosomal compartment, the hydrolysis of glucosylceramides/GlcCers into free ceramides and glucose | 1.082887 |
| CERS5 | Ceramide Synthase 5 | Ceramide synthase that catalyzes the transfer of the acyl chain from acyl-CoA to a sphingoid base, with high selectivity toward palmitoyl-CoA (hexadecanoyl-CoA; C16:0-CoA) | 1.07968 |
| MPD3 | Myopathy, Distal 3 | Diseases associated with MPD3 include Myopathy, Distal, 3 and Miyoshi Muscular Dystrophy | 0.5974959 |
| SPTLC3 | Serine Palmitoyltransferase 3 | Serine palmitoyltransferase (SPT) | 0.6453416 |
| CERK | Ceramide Kinase | Catalyzes specifically the phosphorylation of ceramide to form ceramide 1-phosphate | 1.120135 |
| GBA2 | Glucosylceramidase Beta 2 | Non-lysosomal glucosylceramidase that catalyzes the hydrolysis of glucosylceramides/GlcCers to free glucose and ceramides | 1.297342 |
| SMPD4 | Sphingomyelin Phosphodiesterase 4 | Catalyzes the hydrolysis of membrane sphingomyelin to form phosphorylcholine and ceramide | 1.305204 |
| UGCG | UDP-Glucose Ceramide Glucosyltransferase | Participates in the initial step of the glucosylceramide-based glycosphingolipid/GSL synthetic pathway | 0.8333344 |
| CERS3 | Ceramide Synthase 3 | Ceramide synthase that catalyzes the transfer of the acyl chain from acyl-CoA to a sphingoid base, with high selectivity toward very- and ultra-long-chain fatty acyl-CoA (chain length greater than C22) | 0.169936 |
| CERS6 | Ceramide Synthase 6 | Ceramide synthase that catalyzes the transfer of the acyl chain from acyl-CoA to a sphingoid base, with high selectivity toward palmitoyl-CoA (hexadecanoyl-CoA; C16:0-CoA) | 0.4807154 |


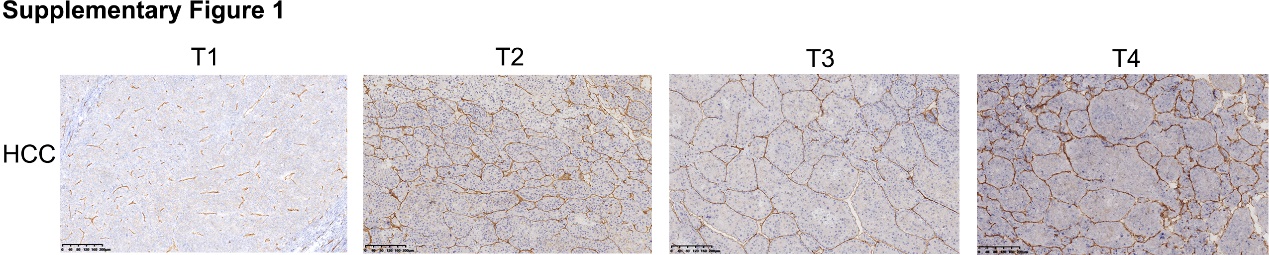


**Supplementary Figure 1.** **S1PR1 is upregulated in the vasculature of HCC tissues at an advanced stage.**

Representative pictures of the expression of S1PR1 in the vasculature of HCC tissues with different T stages according to TNM stages. Scale bar: 200 μm.


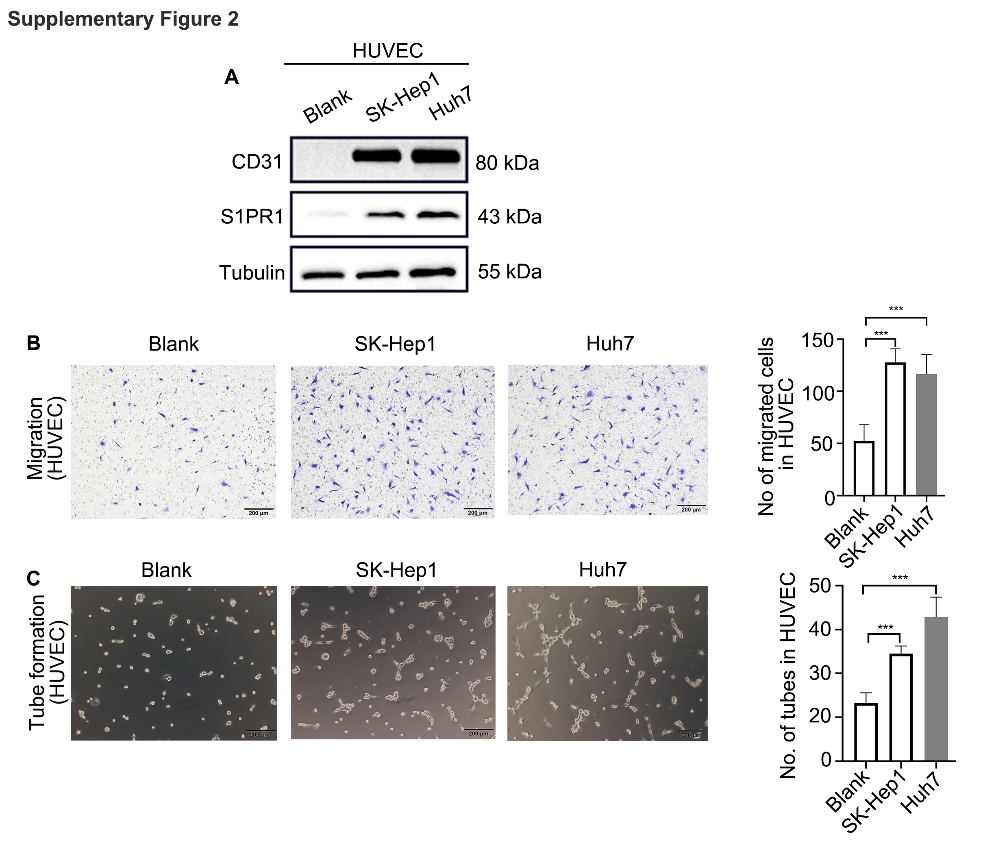


**Supplementary Figure 2. Condition medium from SK-Hep1 and Huh7 cells promotes S1PR1 expression and angiogenesis in HUVECs.**

(A) The expression of CD31 and S1PR1 was detected in HUVECs by WB after treatment with SK-Hep1 and Huh7 cell conditioned medium for 48 h.

(B-C) Migration (B) and tube formation (C) were detected in HUVECs after treatment with SK-Hep1 and Huh7 cell conditioned medium for 48 h. ***p<0.001, scale bar: 200 μm.


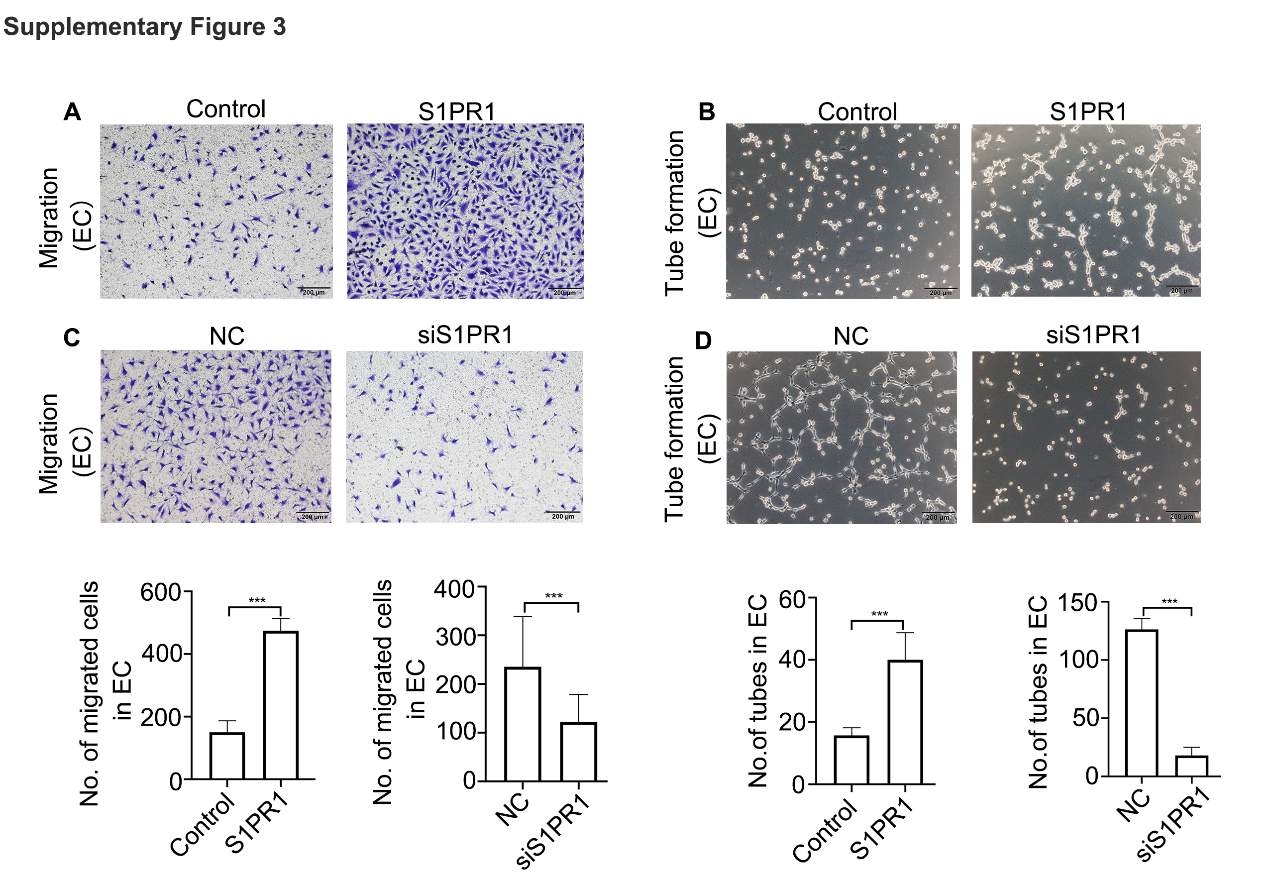


**Supplementary Figure 3. Overexpression of S1PR1 promotes EC migration and tube formation.**

(A-B) The migration and tube formation capacities of ECs were assessed when S1PR1 was overexpressed. Scale bar: 200 μm.

(C-D) The migration and tube formation capacities of ECs were assessed when S1PR1 was downregulated. Scale bar: 200 μm.


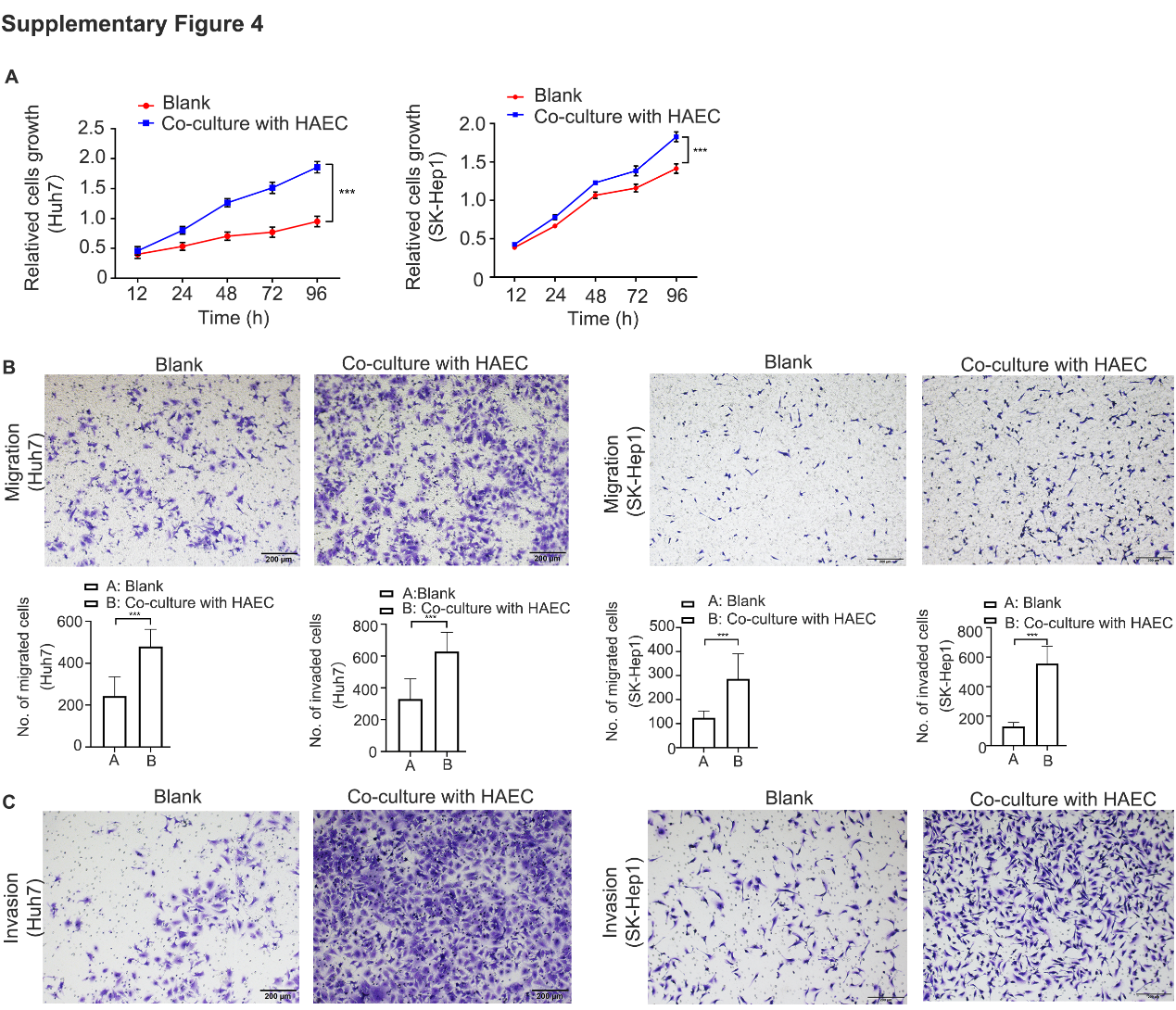


**Supplementary Figure 4**. **The proliferation, invasion, and migration of HCC cells were promoted when the cells were co-cultured with HAECs.**

(A) The proliferation of HCC cells (Huh7 and SK-Hep1) after coculture with HAECs for 48 h was determined by CCK-8 assay.

(B-C) The migration and invasion of HCC cells (Huh7 and SK-Hep1) after coculture with HAECs for 48 h were determined. Scale bar: 200 μm.


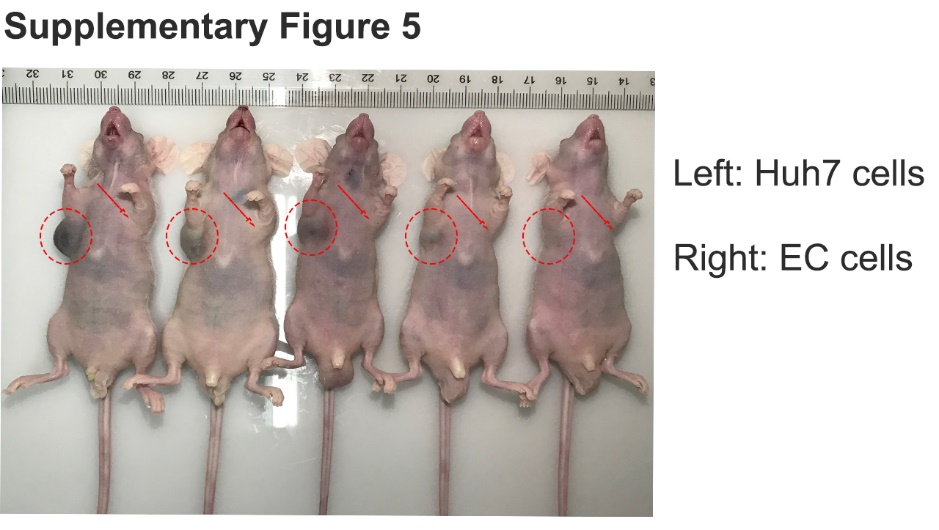


**Supplementary Figure 5. ECs cannot form xenografts in nude mice.**

Representative images of xenografts of Huh7 liver cancer cells in the nude mice (right flank) at 28 days; ECs could not form xenografts in the nude mice (left flank).


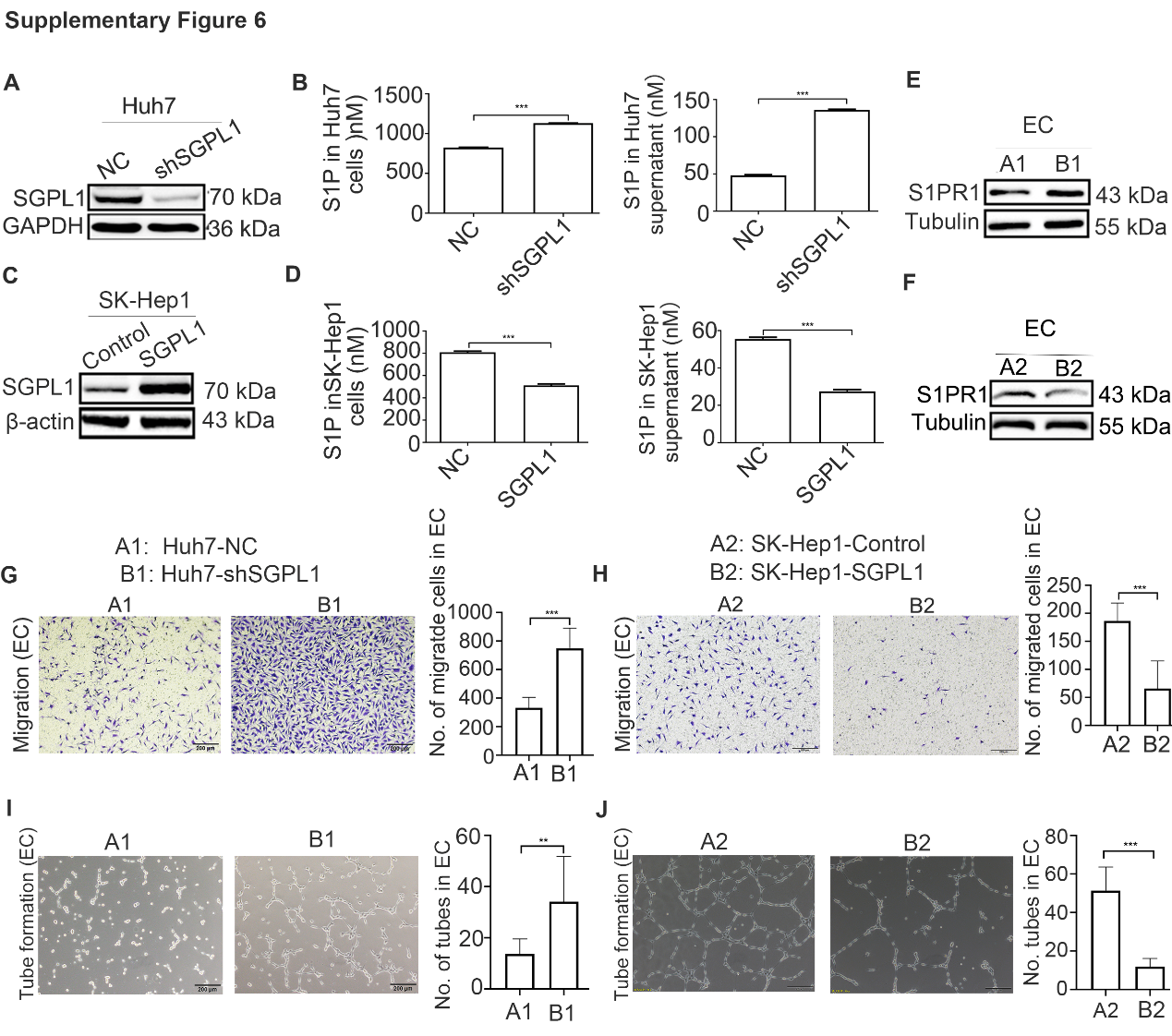


**Supplementary Figure 6. S1P promotes S1PR1 expression and angiogenesis in HAECs.**

(A and C) WB was used to detect SGPL1 expression in Huh7 cells stably expressing SGPL1-shRNA (A) and in SK-Hep1 cells stably overexpressing SGPL1 (C).

(B and D) The content of S1P in cells or supernatant of Huh7 cells with SGPL1 downregulation (B) or SK-Hep1 cells with SGPL1 overexpression (D) was measured by ELISA.

(E-F) S1PR1 in ECs treated with conditioned media from Huh7 cells with SGPL1 downregulation (E, A1: NC, B1: shSGPL1) or SK-Hep1 cells with SGPL1 overexpression (F, A2: Control, B2: SGPL1) was assessed by WB.

(G-J) Migration assays (G-H) and tube formation assays (I-J) were used to detect the function of ECs treated with the supernatant of Huh7-shSGPL1 or SK-SGPL1 cells. Scale bar: 200 μm.

Data are presented as the means ± SDs, and an independent t test was used to analyse the significant difference. ***P < 0.001.


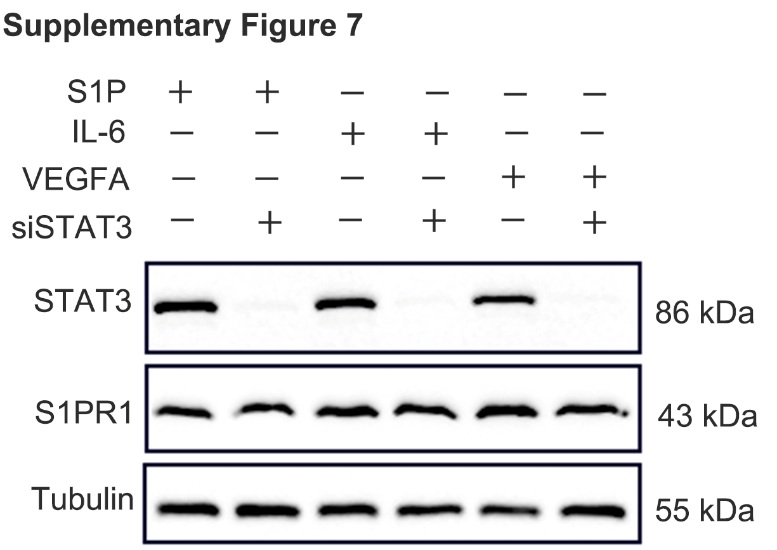


**Supplementary Figure 7. S1P, IL-6, and VEGFA promote the expression of S1PR1 independent of the level of total STAT3.**

The expression of S1PR1 induced by S1P (40 μM), IL-6 (25 ng/ml), and VEGFA (80 ng/ml) in EC cells pretreated with siSTAT3 was detected by WB.


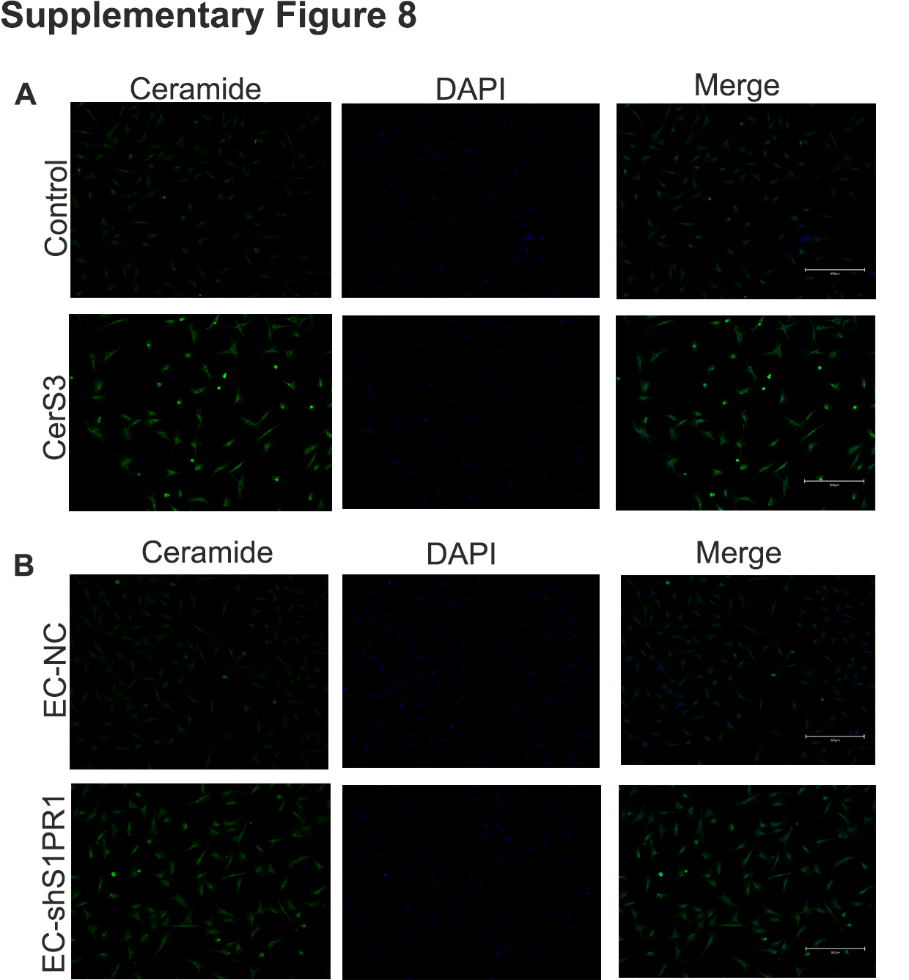


**Supplementary Figure 8. The level of ceramide was regulated by CerS3 and S1PR1 in ECs.**

(A-B) The expression of ceramides (green) in ECs treated with CerS3 plasmid and shS1PR1 was determined by IF. DAPI staining is indicated in blue. Scale bar: 150 μm.


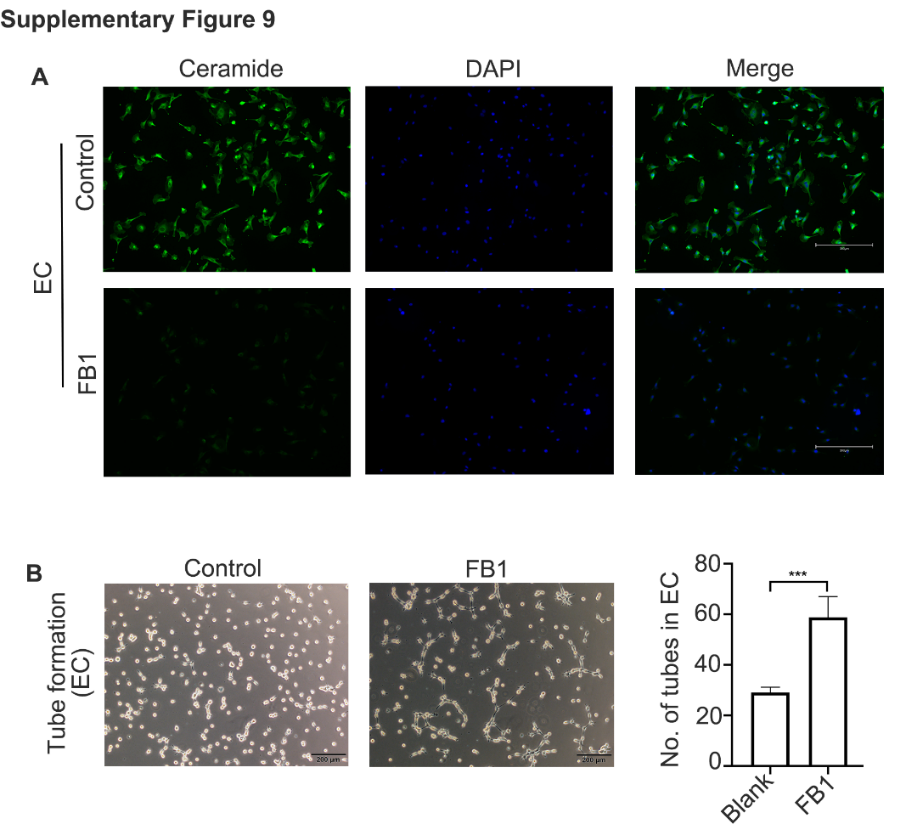


**Supplementary Figure 9. FB1 decreases the level of ceramides and increases the tube formation capacity in ECs.**

(A) The level of ceramide in ECs after treatment with FB1(0.1 μM) for 48 h was detected by IF. Scale bar: 150 μm.

(B) The tube formation capacity of ECs after treatment with FB1(0.1 μM) for 48 h was detected. ***p<0.01, Scale bar: 200 μm.

1. Alam MS. Proximity Ligation Assay (PLA). Curr Protoc Immunol. 2018;123(1):e58.
