# Supplementary figures and images for: S1PR1 induces metabolic reprogramming of ceramide in vascular endothelial cells, affecting hepatocellular carcinoma angiogenesis and progression
Source: Cell Death Dis. 2022 Sep 6;13(9):768. doi: 10.1038/s41419-022-05210-z (PMC9448762; doi:10.1038/s41419-022-05210-z)

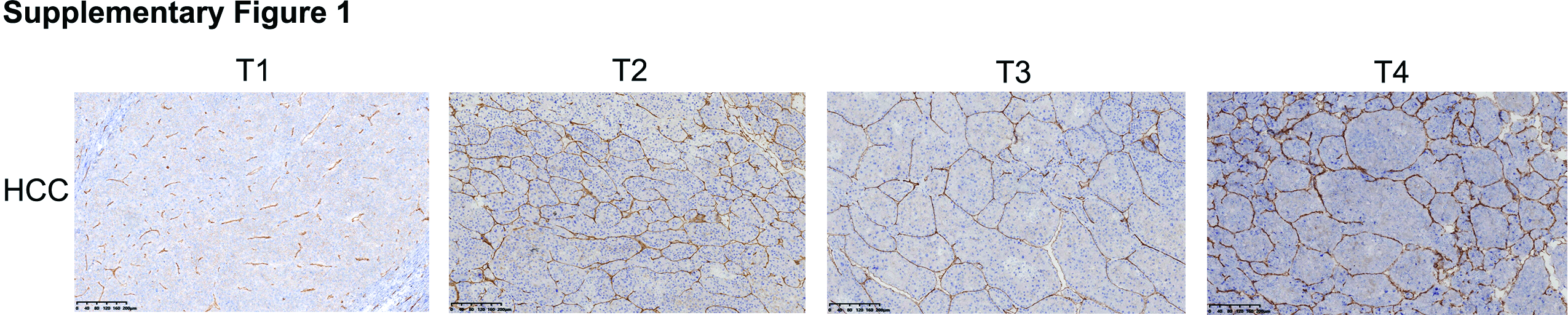

Supplement: Supplementary file 2 — Figure S1 [file 41419_2022_5210_MOESM2_ESM.tif]

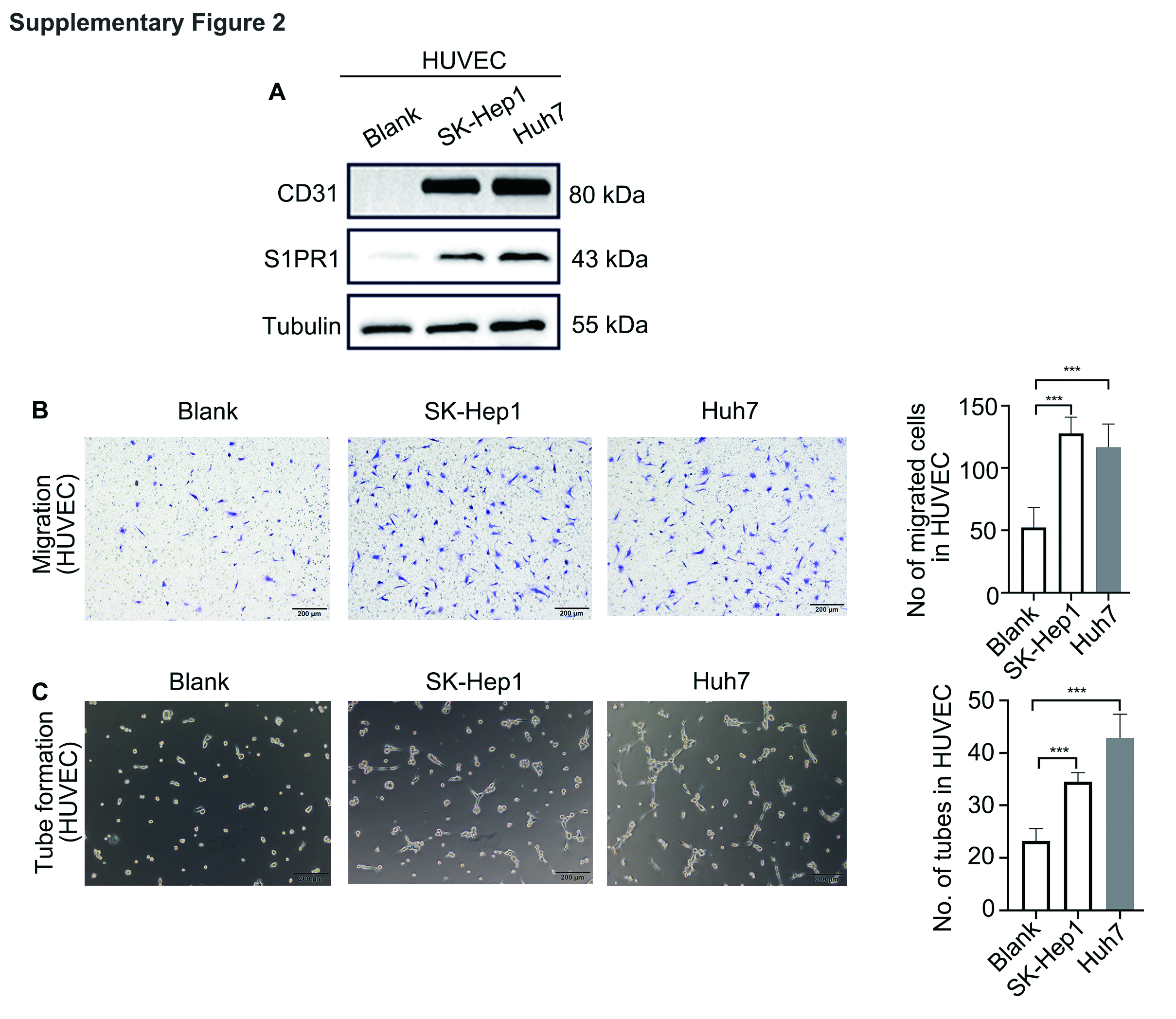

Supplement: Supplementary file 3 — Figure S2 [file 41419_2022_5210_MOESM3_ESM.tif]

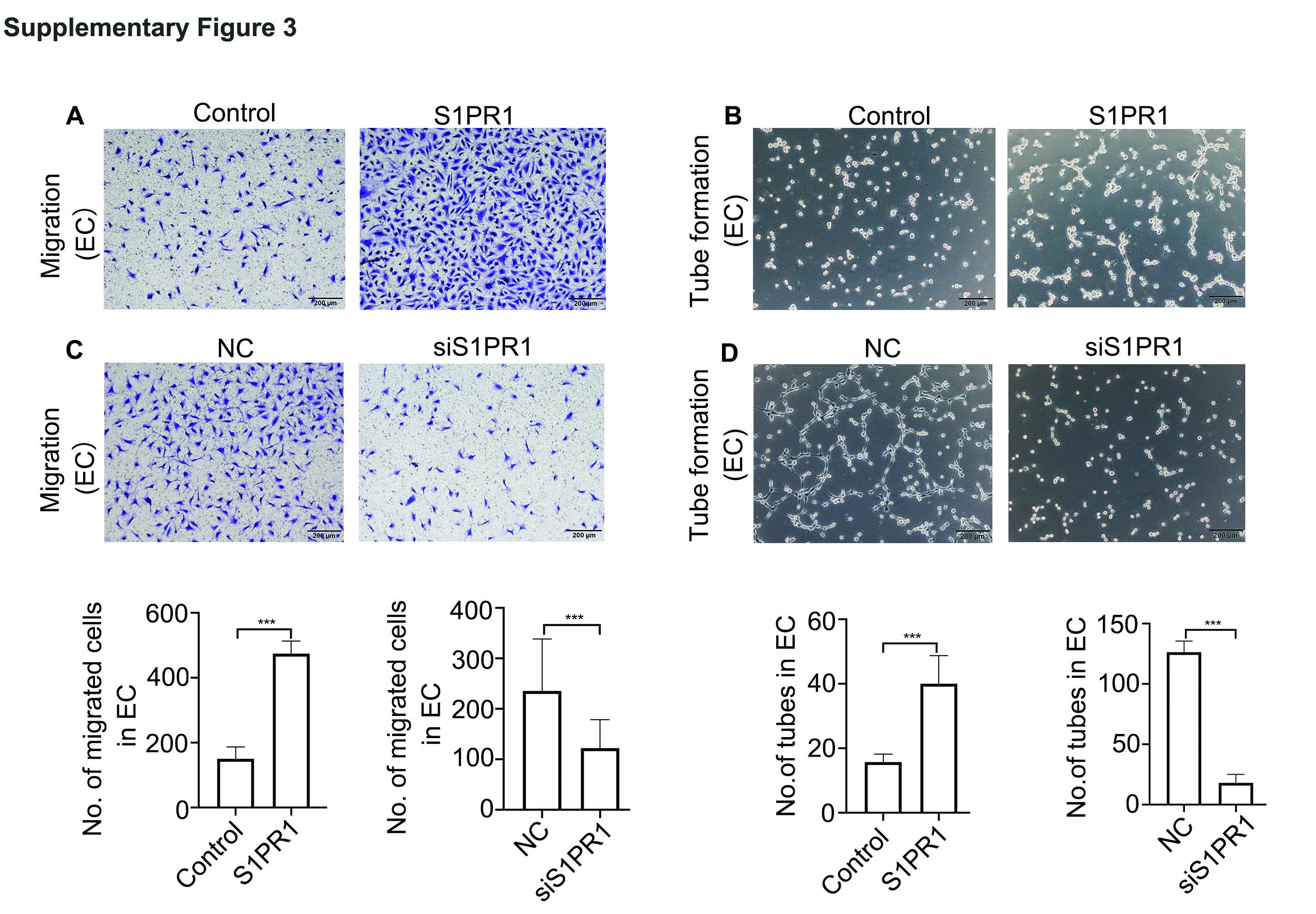

Supplement: Supplementary file 4 — Figure S3 [file 41419_2022_5210_MOESM4_ESM.tif]

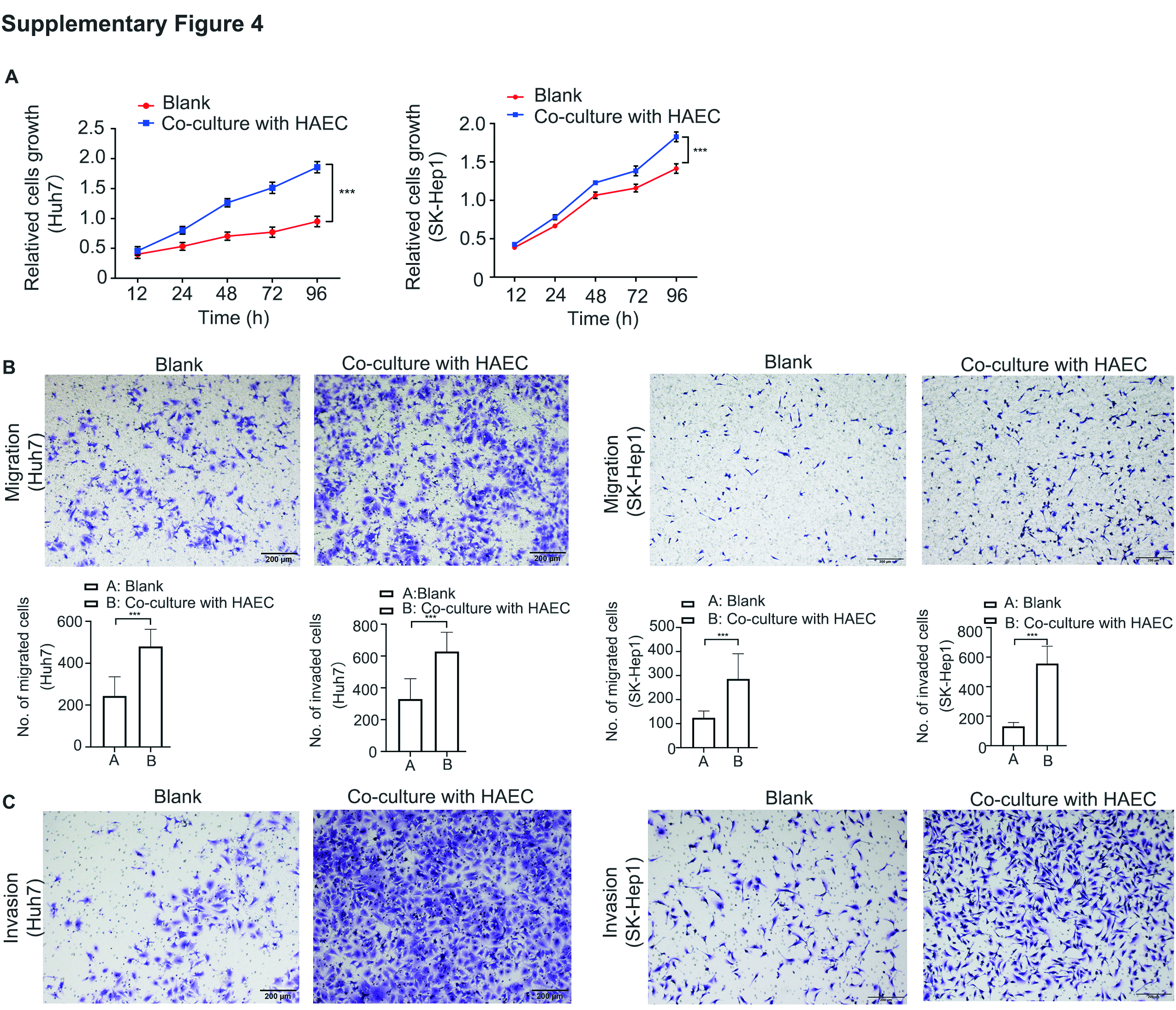

Supplement: Supplementary file 5 — Figure S4 [file 41419_2022_5210_MOESM5_ESM.tif]

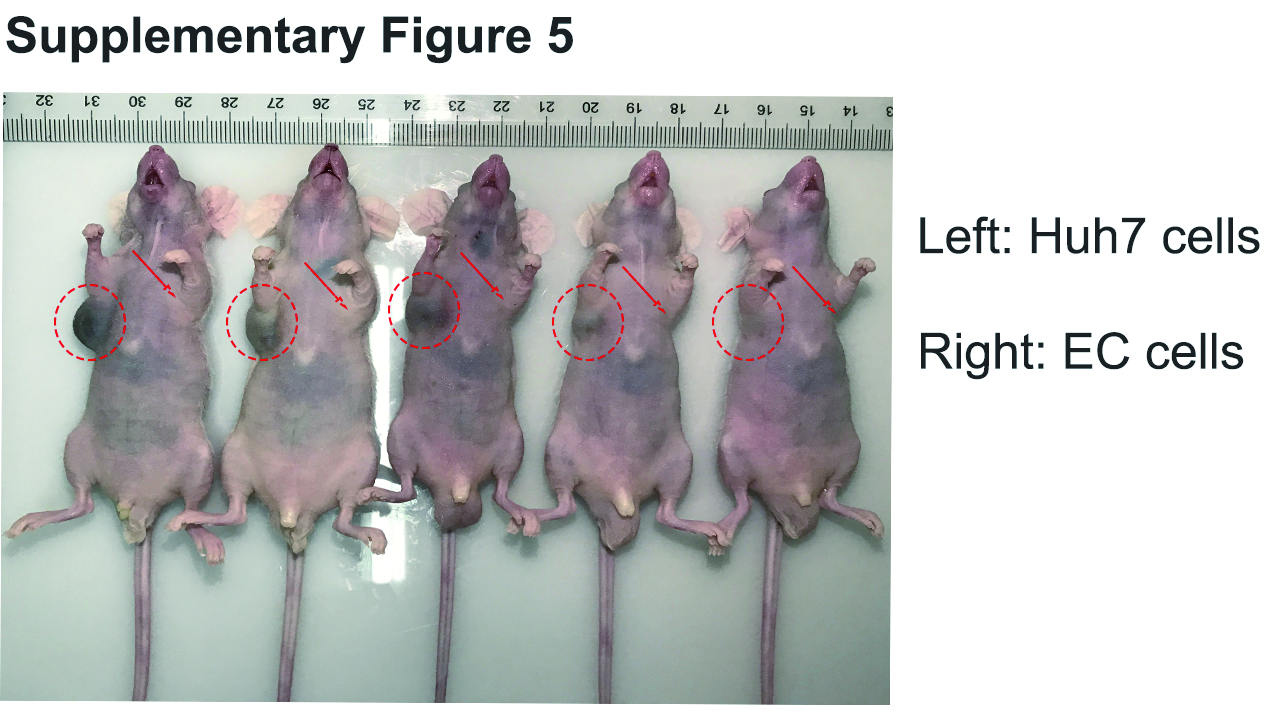

Supplement: Supplementary file 6 — Figure S5 [file 41419_2022_5210_MOESM6_ESM.tif]

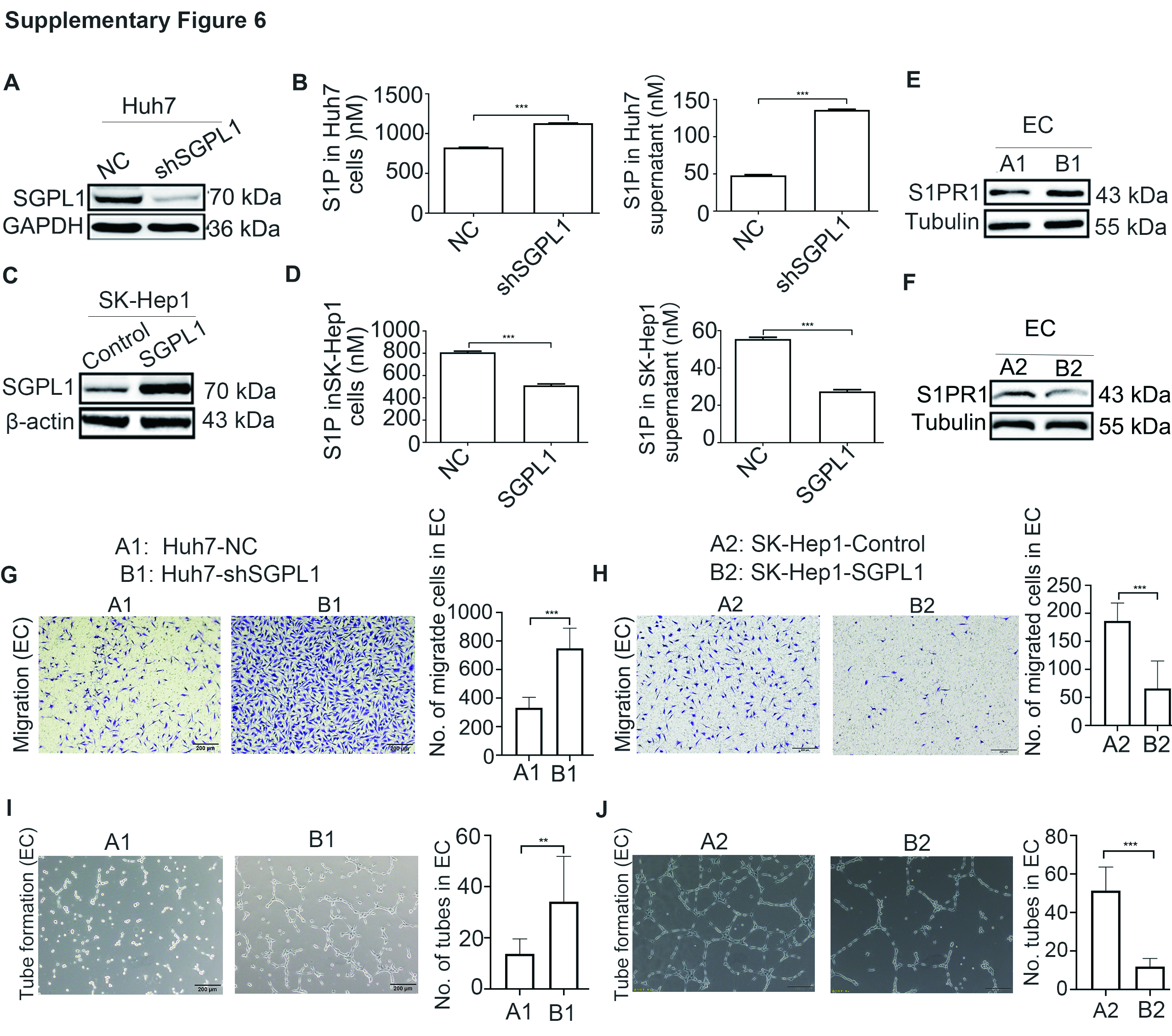

Supplement: Supplementary file 7 — Figure S6 [file 41419_2022_5210_MOESM7_ESM.tif]

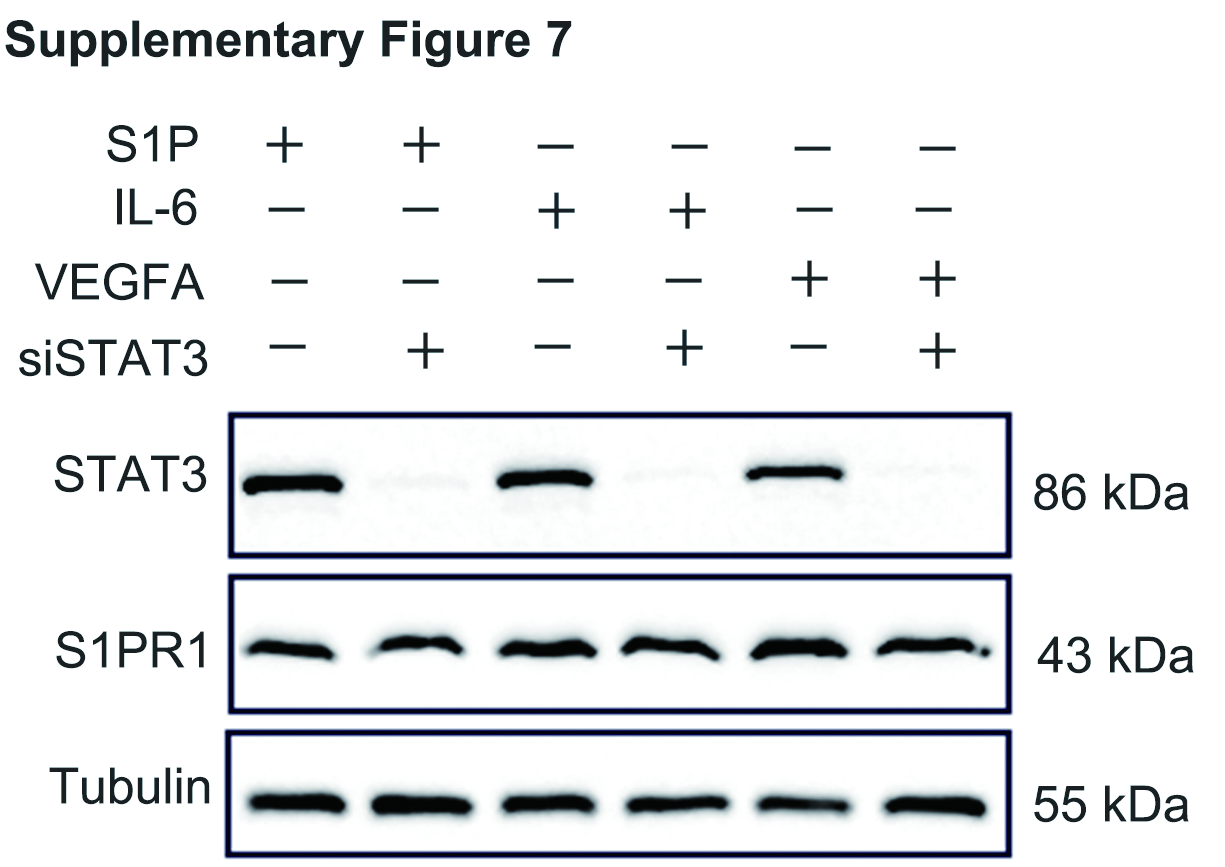

Supplement: Supplementary file 8 — Figure S7 [file 41419_2022_5210_MOESM8_ESM.tif]

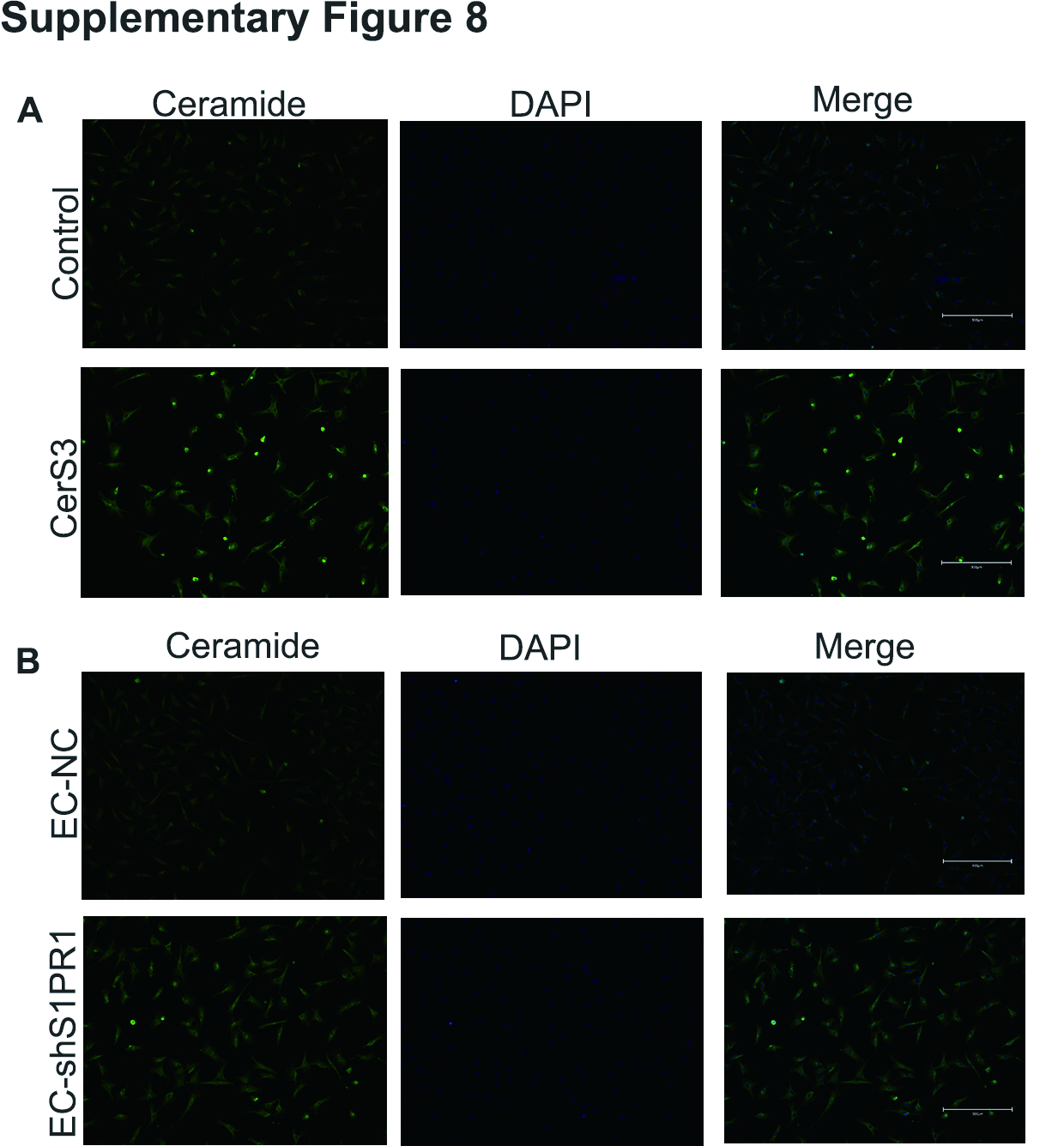

Supplement: Supplementary file 9 — Figure S8 [file 41419_2022_5210_MOESM9_ESM.tif]

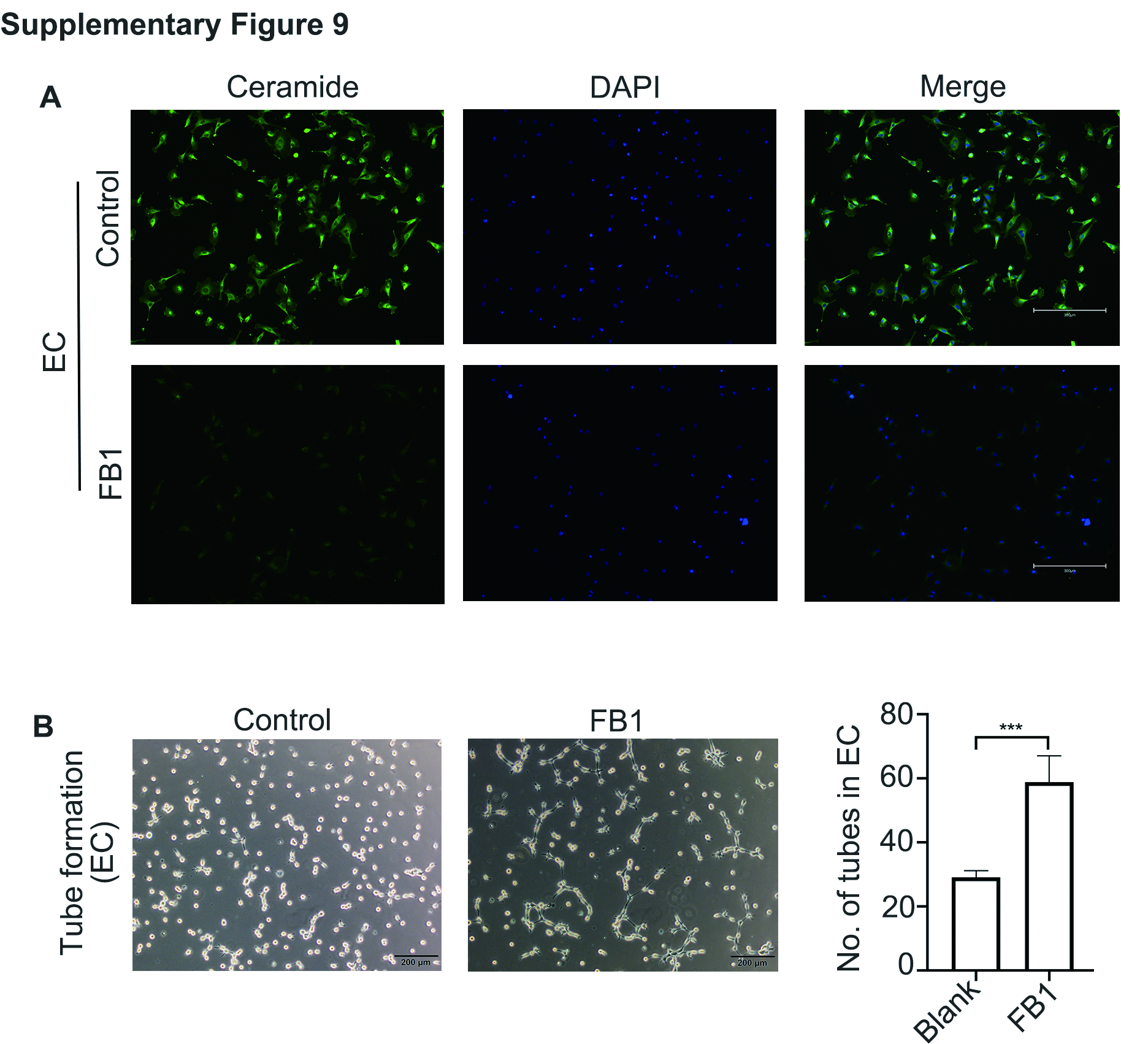

Supplement: Supplementary file 10 — Figure S9 [file 41419_2022_5210_MOESM10_ESM.tif]
