# Supplementary material for: S1PR1 induces metabolic reprogramming of ceramide in vascular endothelial cells, affecting hepatocellular carcinoma angiogenesis and progression
Source: Cell Death Dis. 2022 Sep 6;13(9):768. doi: 10.1038/s41419-022-05210-z (PMC9448762; doi:10.1038/s41419-022-05210-z)

Result 2. The functions of HAECs induced by conditioned media from HCC cells (SK-Hep1 and Huh7) are enhanced.

(1). The markers of ECs CD31/CD34/CD105 and S1PR1 in HAECs induced by conditioned media from SK-Hep1 cells were assessed by WB.

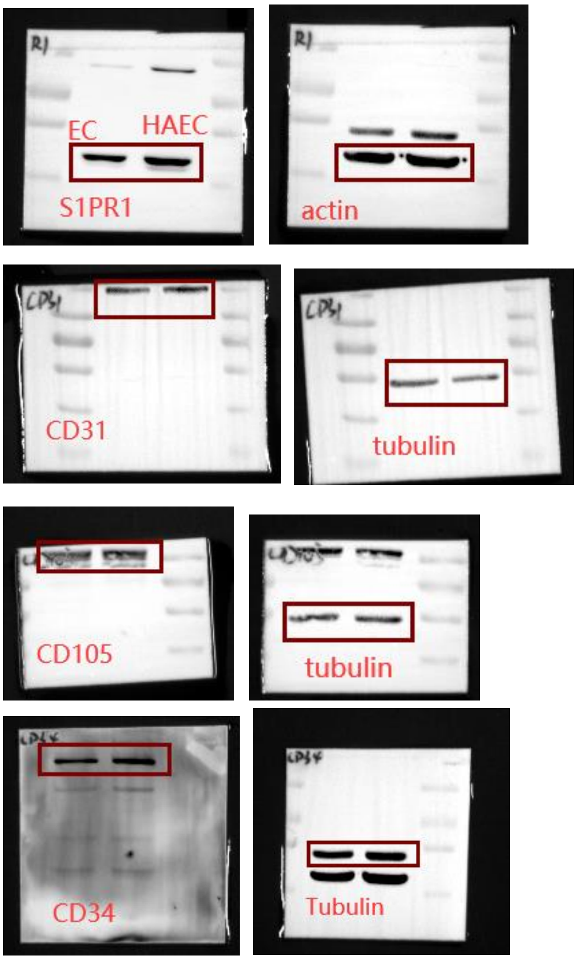

(2). The markers of ECs CD31/CD34/CD105 and S1PR1 in HAECs induced by conditioned media from Huh7 cells were assessed by WB.

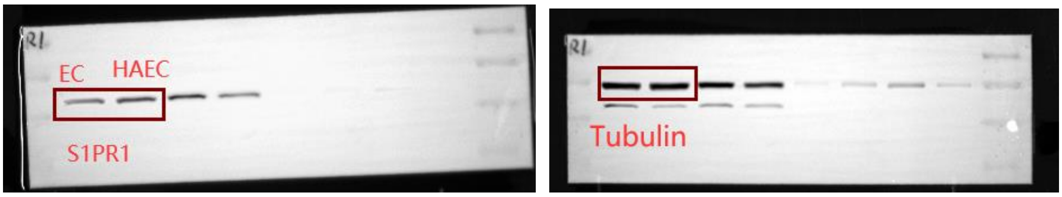

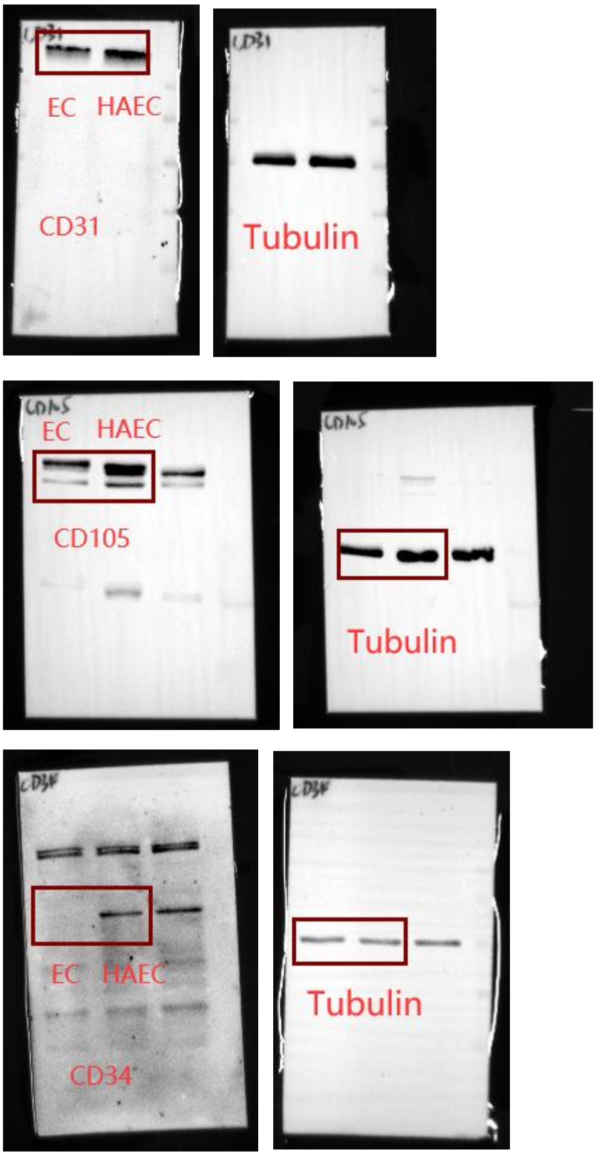

Result 4. S1PR1 was upregulated in HAECs via the phosphorylation of STAT3, which was stimulated by the conditioned media from HCC cells.

(1). The protein expression of S1PR1 in EC treatment with 40 μM S1P for 48 h was tested by WB.

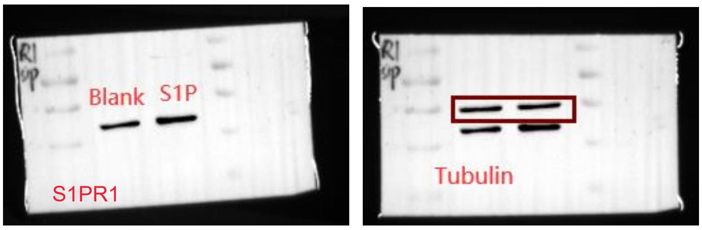

(2). STAT3 phosphorylation (Y705) in EC treatment with 40 μM S1P for 48 h was tested by WB.

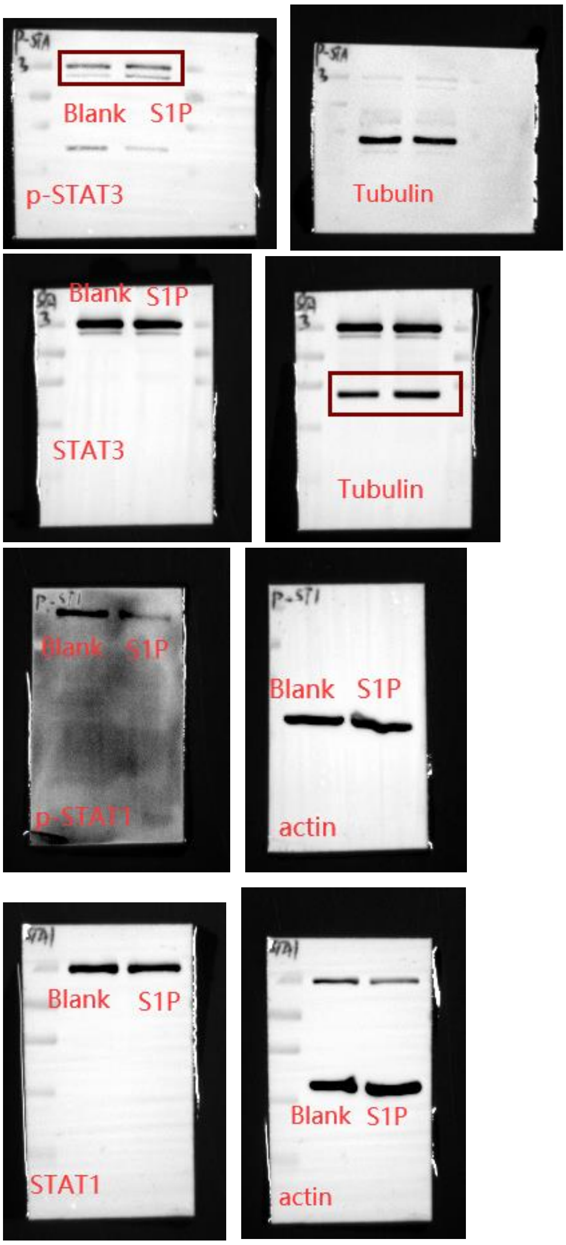

(3). STAT1 and STAT3 phosphorylation (Y705) in EC and HAECs was detected by WB.

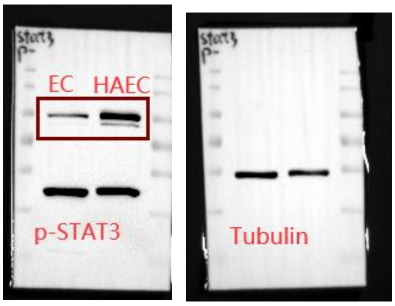

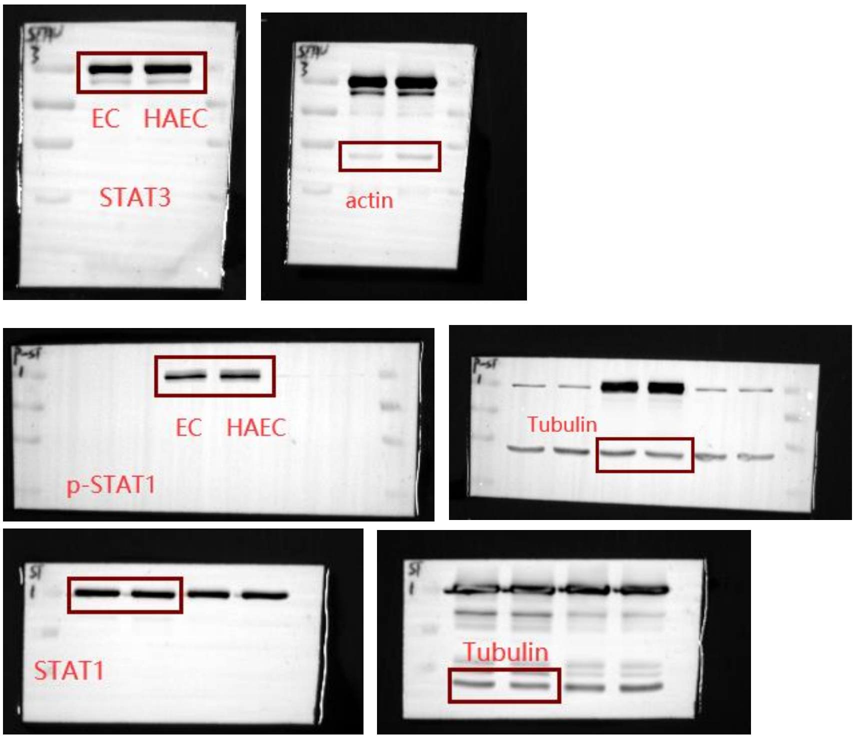

(4). STAT3 phosphorylation (Y705) in EC treatment with 80 ng/mL VEGFA for 48 h was tested by WB.

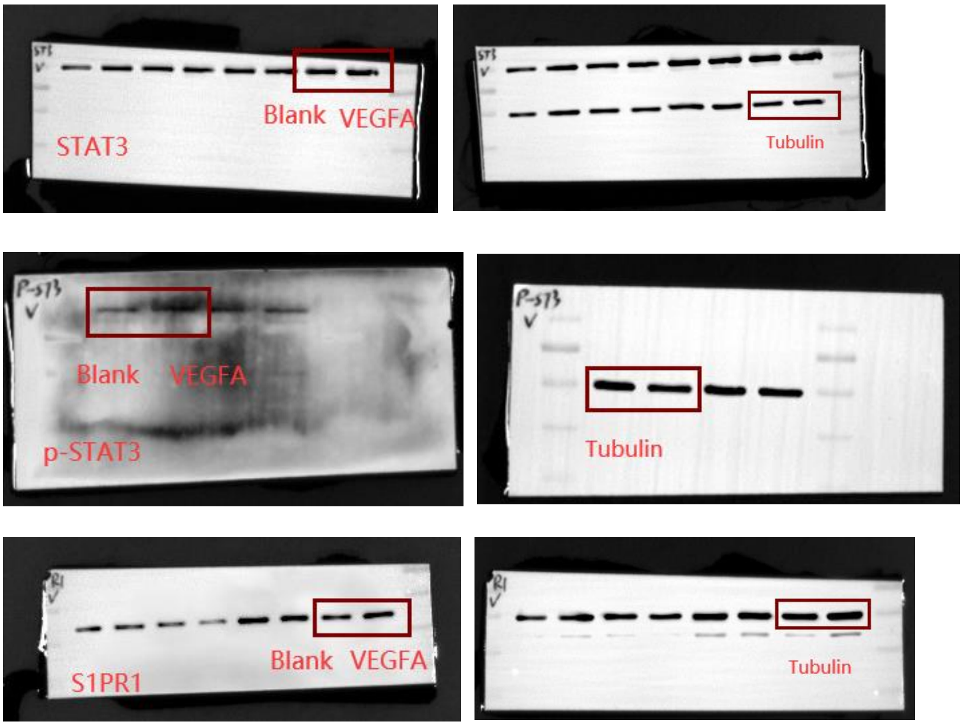

(5). STAT3 phosphorylation (Y705) in EC treatment with 25 ng/mL IL-6 for 48 h was tested by WB.

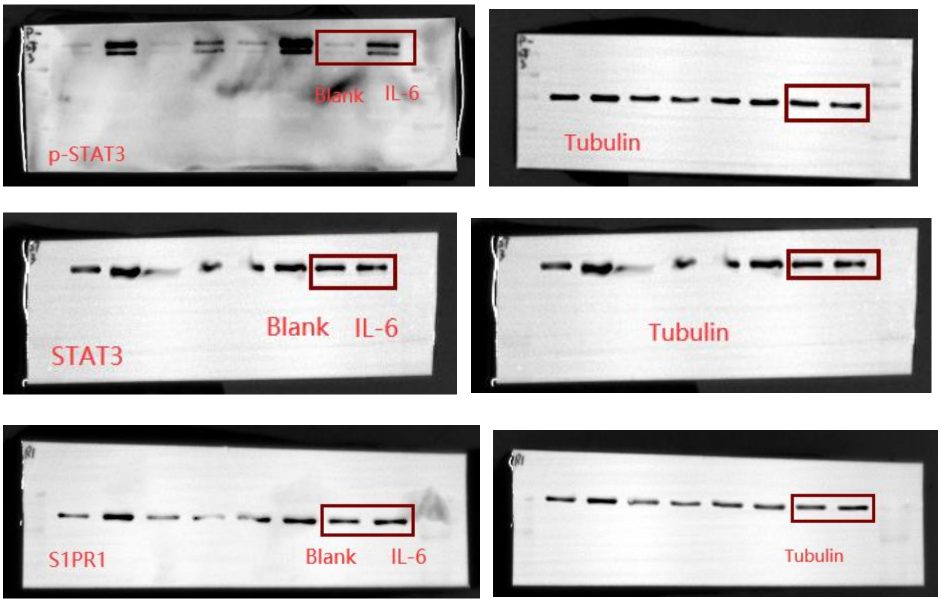

(6). The expression of S1PR1 in HAECs treated with stattic (an inhibitor of STAT3 phosphorylation at Y705) after pretreatment with IL-6, VEGFA, and S1P was detected by WB.

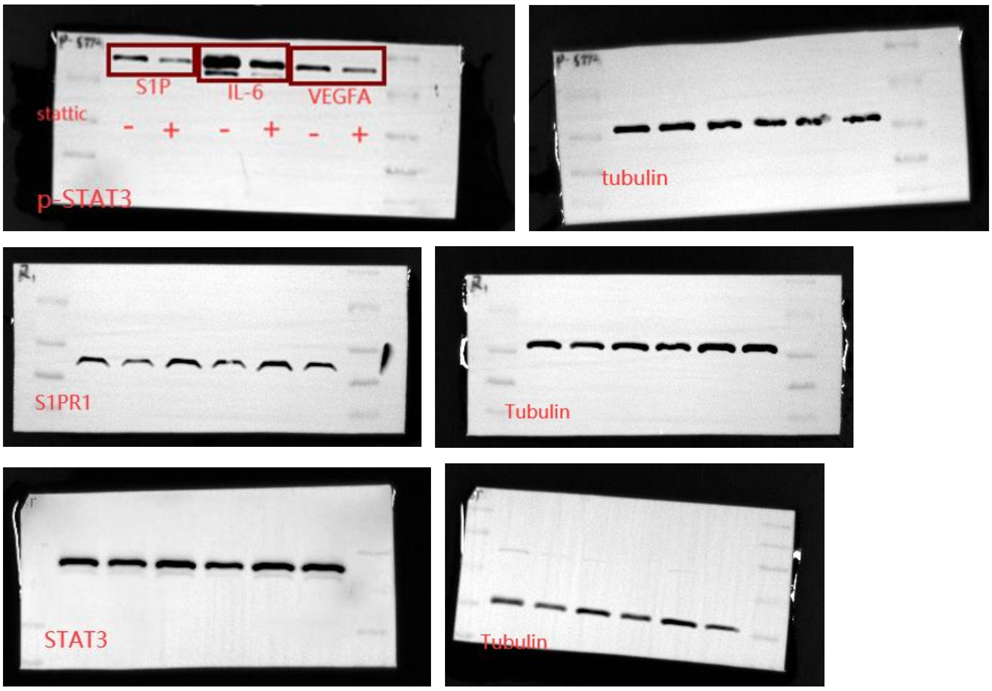

Result 5. S1PR1 upregulation reduced ceramide level via inhibition of CerS3 expression.

(1). CerS3, CerS6, SPHK1, S1PR1 in HAECs was tested by WB.

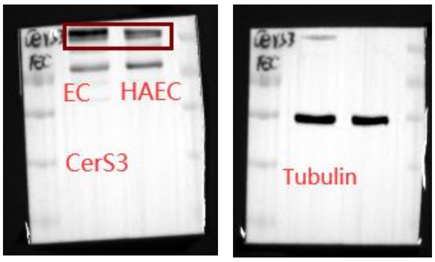

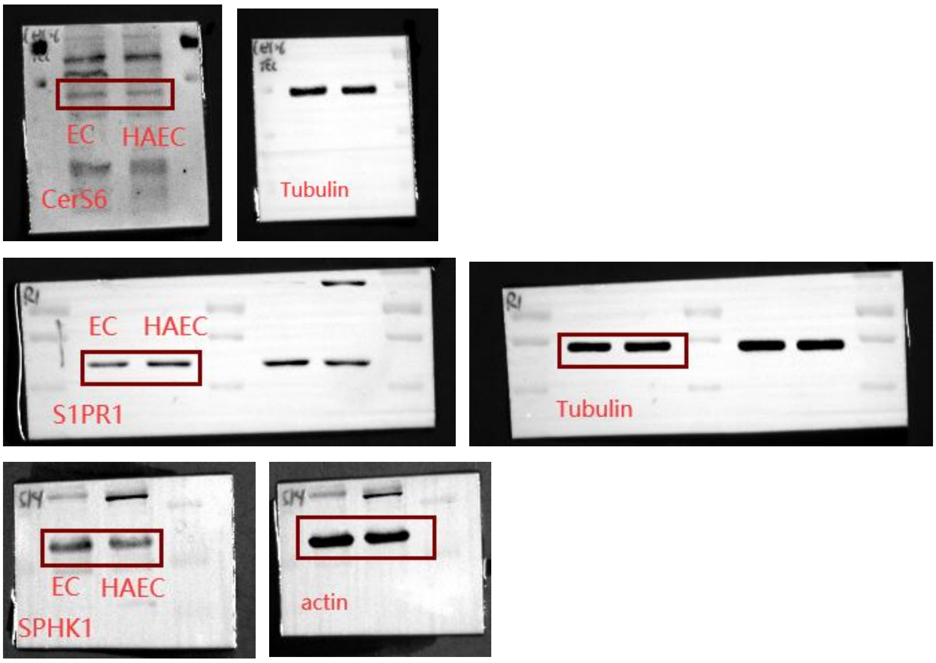

(2). The expression of CerS3 in ECs-shS1PR1 was detected by WB.

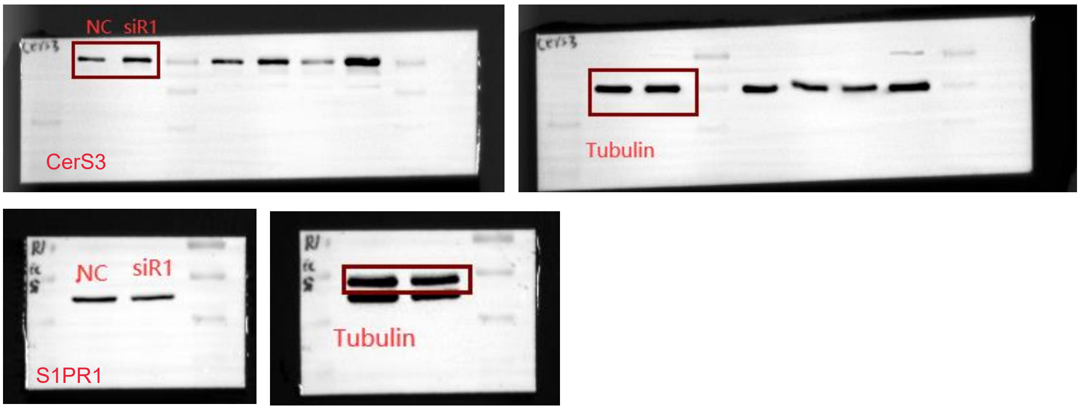

(3). S1P-relatived enzymes SPHK1, SPHK2, SGPL1 in HAECs was tested by WB.

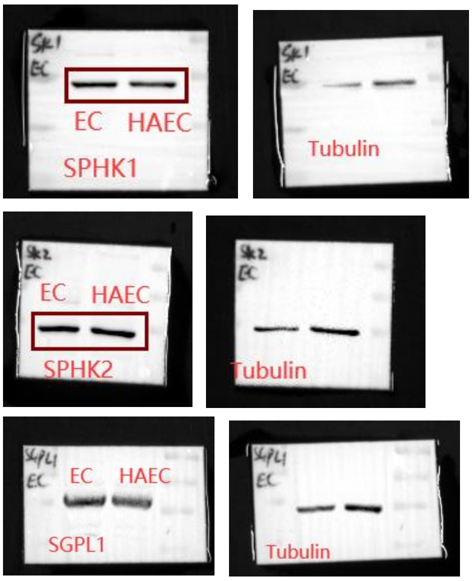

Result 6. A reduction in ceramide levels and its interaction with PTEN activates AKT/ERK signalling to promote angiogenesis.

(1). p-AKT, AKT, p-ERK, ERK and PTEN expression levels in HAECs were performed by WB.

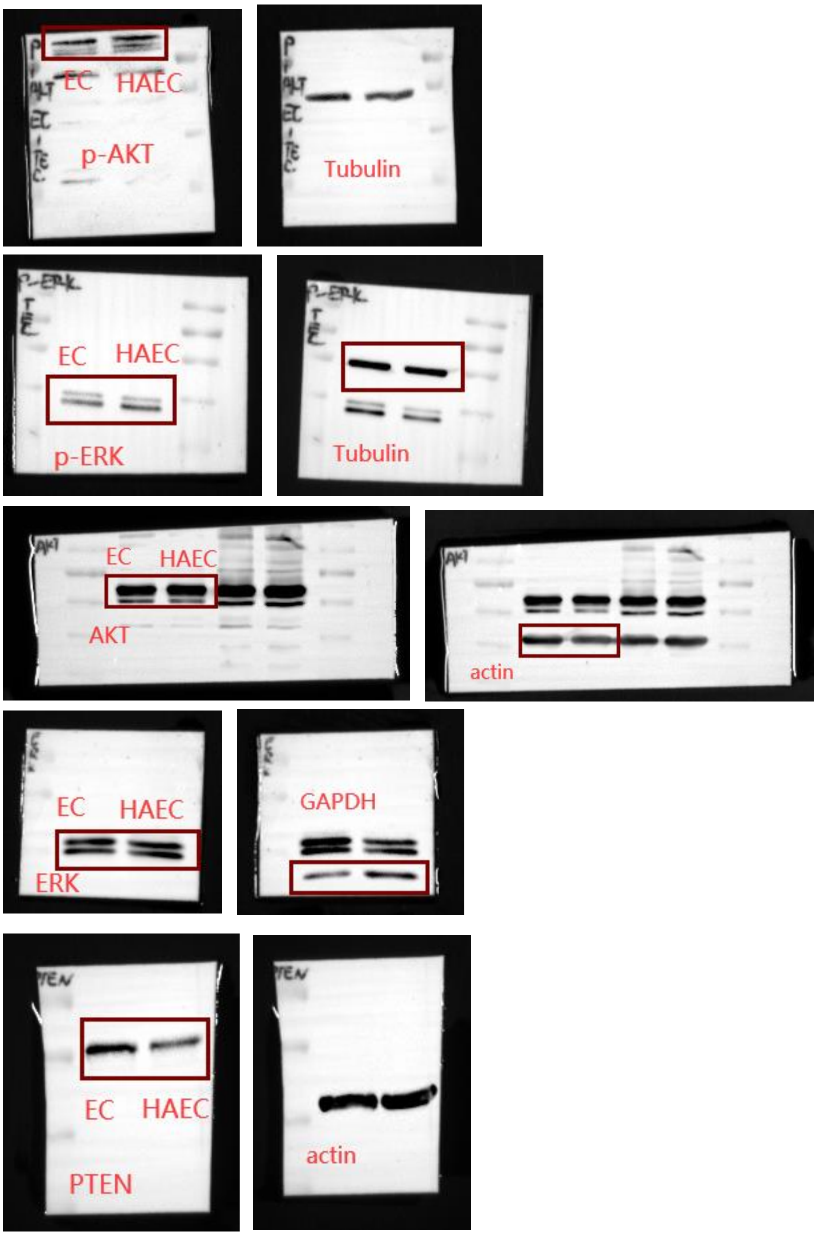

(2). p-AKT, AKT, p-ERK, ERK and PTEN expression levels in EC-shS1PR1 were performed by WB.

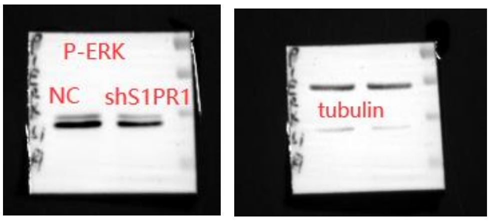

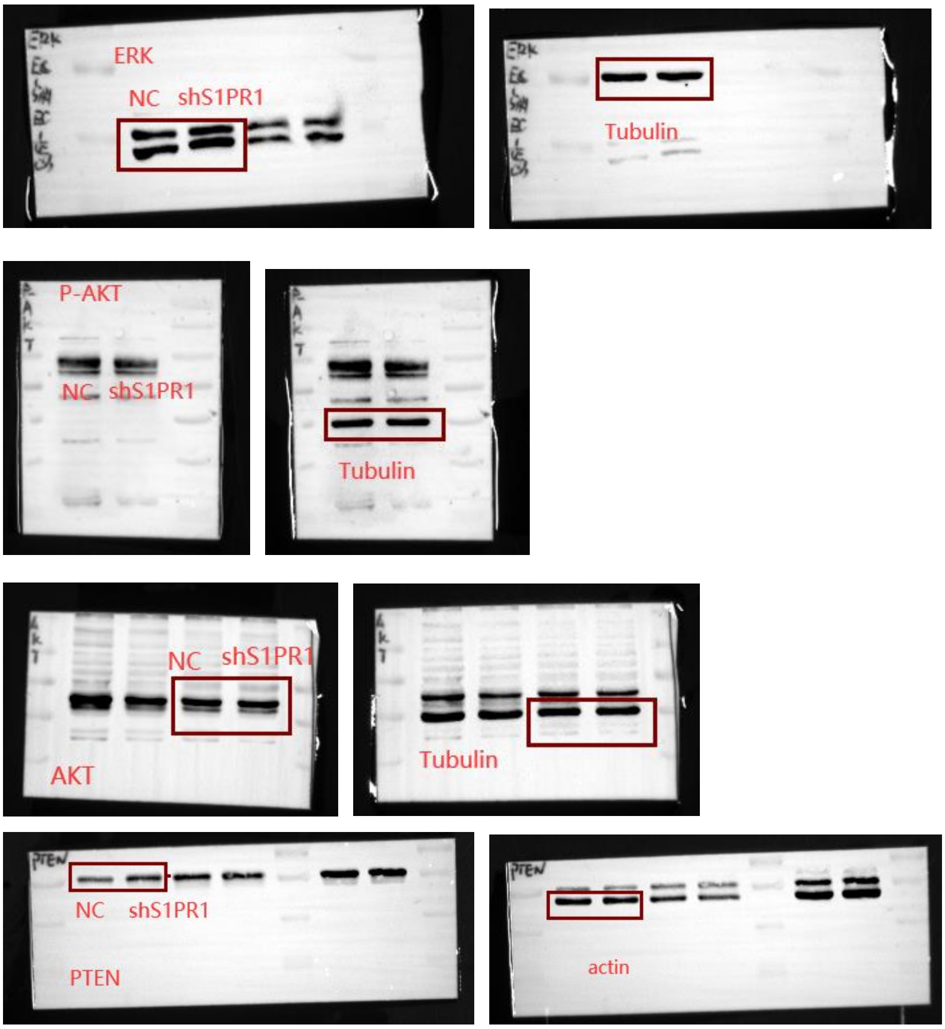

(3). p-AKT, AKT, p-ERK, ERK and PTEN expression levels in EC transfected with plasmid of CerS3 overexpression were performed by WB.

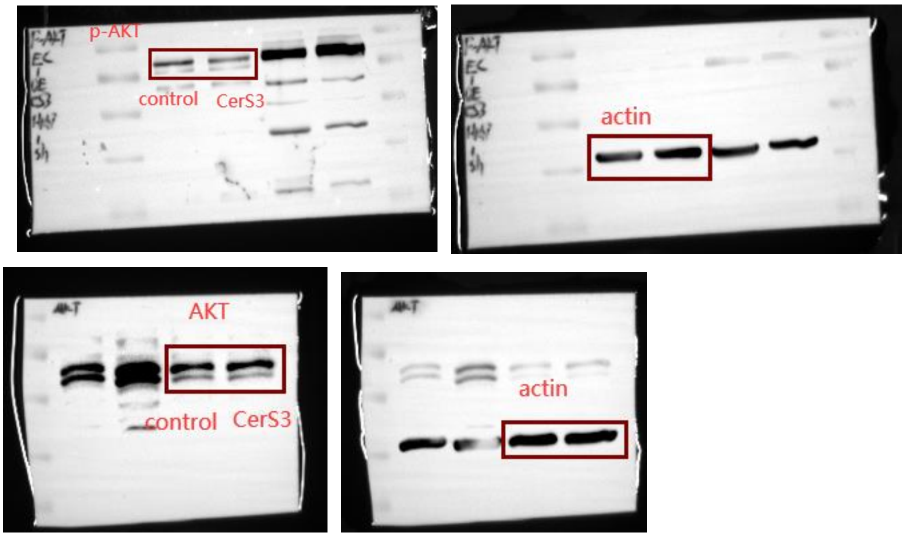

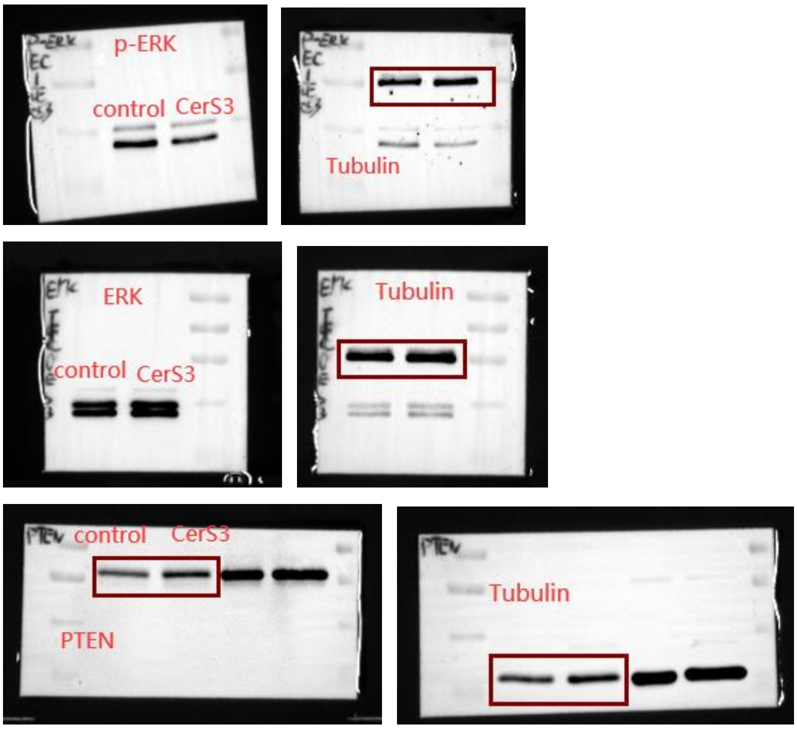

Result 7. S1PR1-induced nuclear translocation of CerS6 inhibits CerS3 expression at the transcriptional level.

(1). The protein expression of CerS2/4/5/6 in cytoplasm and nucleus of HAECs were detected by WB.

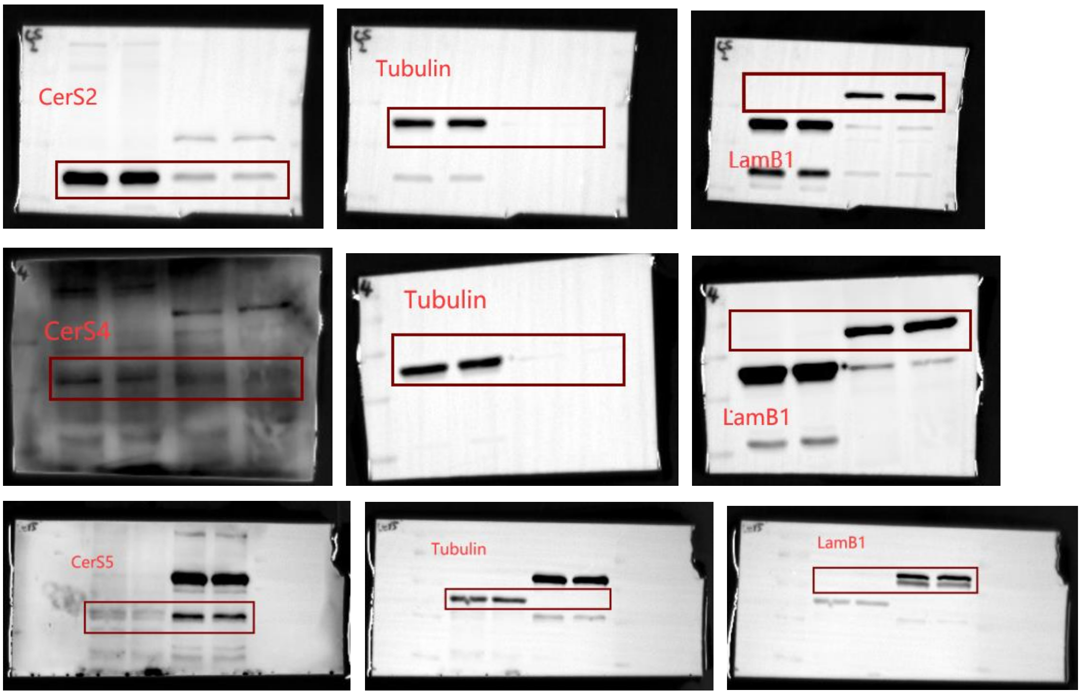

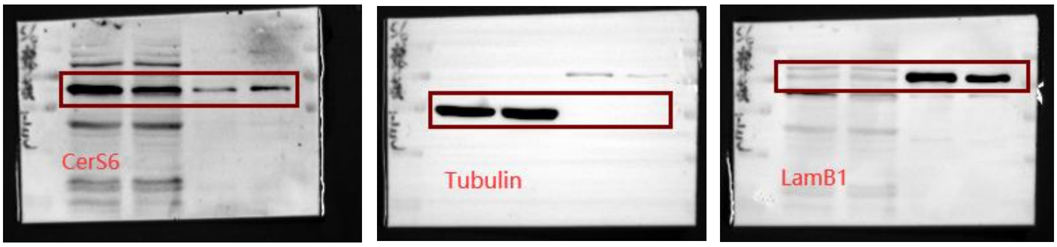

(2). The protein expression of CerS6 in cytoplasm and nucleus of EC-shS1PR1 were tested by WB.

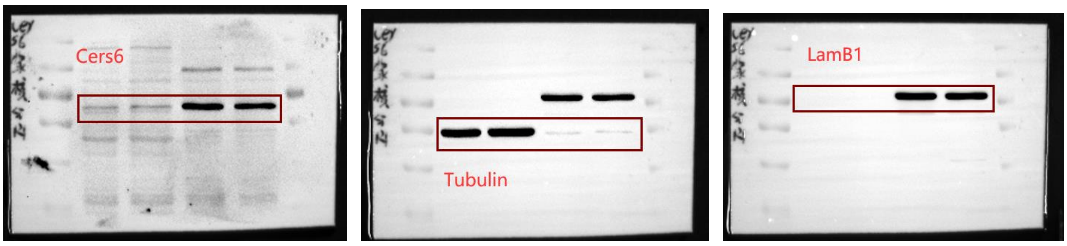

Result 8. High concertation of lenvatinib inhibited S1PR1 and synergistically arrested angiogenesis.

(1) The inhibitory effect of W146 on the promotion of S1PR1 expression induced by the media from SK-Hep1 cells was detected by WB.

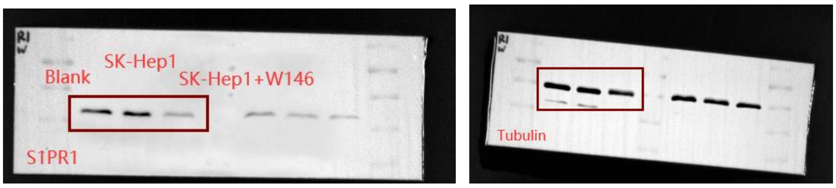

(2). S1PR1 protein expression was tested in EC treated different concentrations (0 – 40 nM).

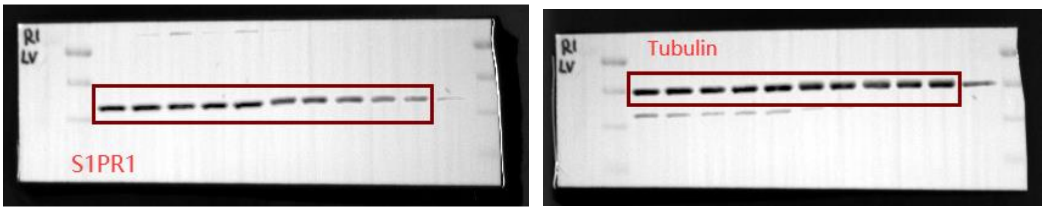

Supplementary Figure 2. The expression of CD31 and S1PR1 was detected in HUVECs by WB after treatment with SK-Hep1 and Huh7 cell conditioned medium for 48 h.

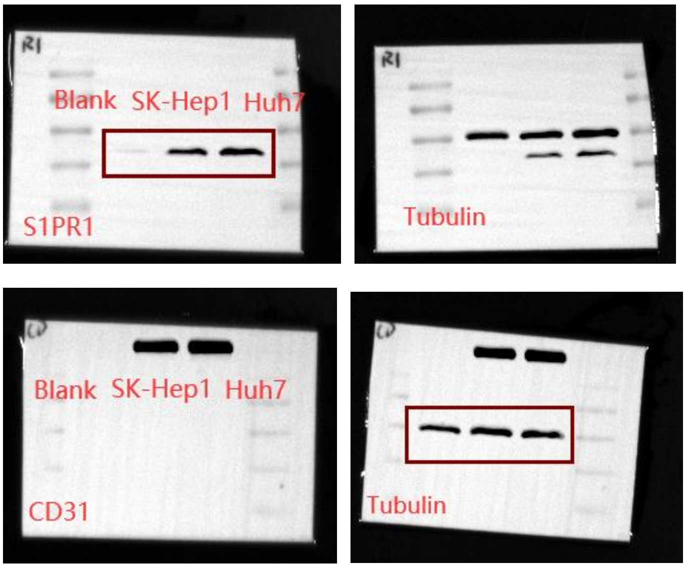

Supplementary Figure 6. S1P increased S1PR1 and promoted angiogenesis of TEC.

(1). WB was used to detected SGPL1 expression in SGPL1-shRNA stably expression in Huh7, and in SGPL1 stably over-expression in SK-Hep1.

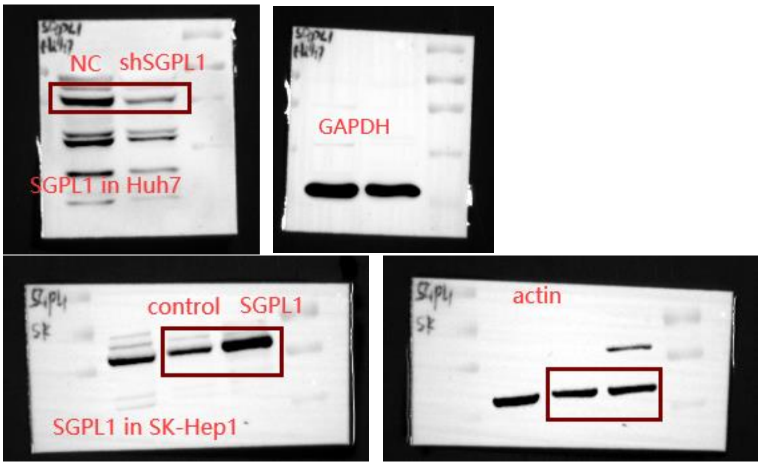

(2). S1PR1 in EC treated with conditioned media from Huh7-shSGPL1 or SK-Hep1-SGPL1 were tested by WB.

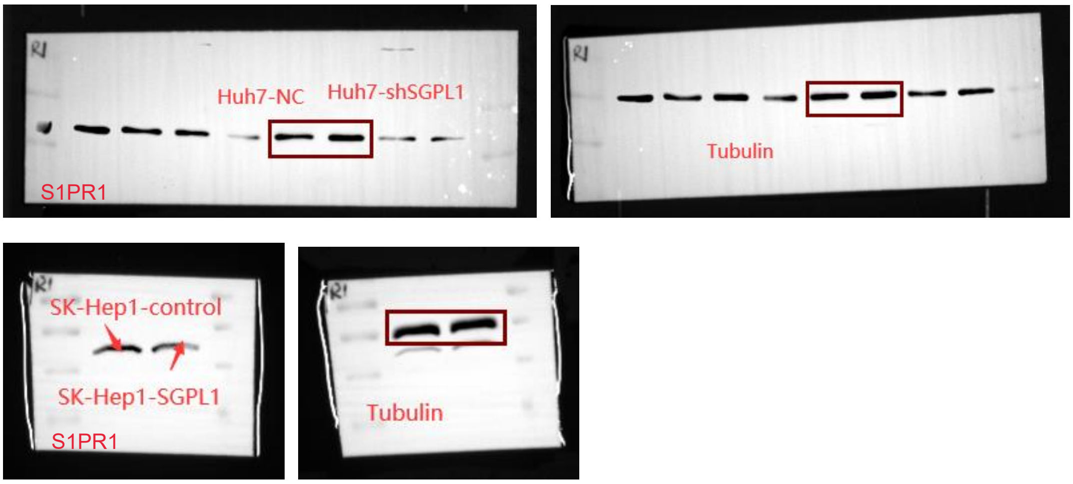

Supplementary Figure 7. The expression of S1PR1 induced by S1P, IL-6, and VEGFA in EC cells pretreated with siSTAT3 was detected by WB.

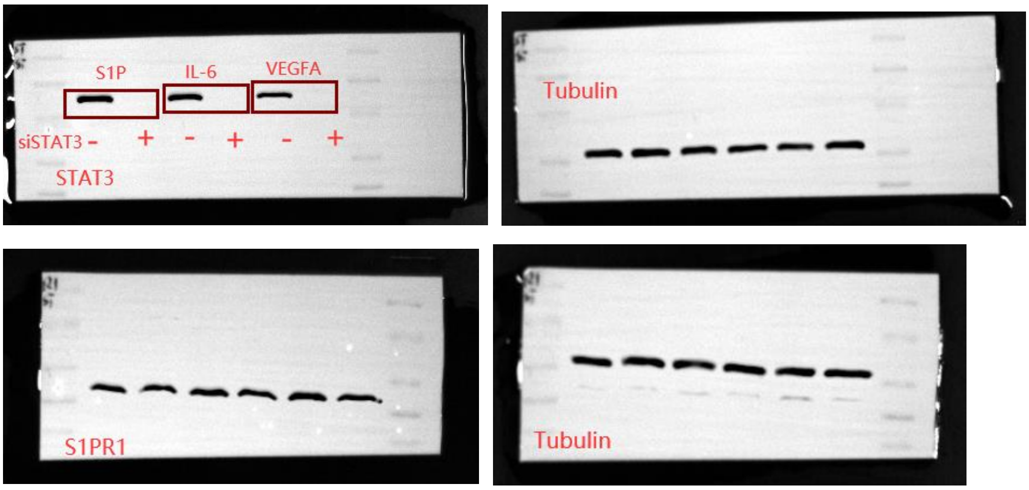

Supplement: Supplementary file 11 — Original Data File [file 41419_2022_5210_MOESM11_ESM.pdf]
